# Hybrid Piezo–Electromagnetic Device Designed to Harvest the Vibrations of the Human Body

**DOI:** 10.3390/mi16060675

**Published:** 2025-05-31

**Authors:** George-Claudiu Zărnescu, Lucian Pîslaru-Dănescu, Ioan Stamatin

**Affiliations:** 1 Laboratory of Sensors/Actuators and Energy Harvesting, National Institute for Research and Development in Electrical Engineering ICPE-CA, 030138 Bucharest, Romania; george.zarnescu@icpe-ca.ro; 2 Faculty of Physics, 3Nano-SAE Research Center, University of Bucharest, Magurele, 077125 Bucharest, Romania; istarom@3nanosae.org

**Keywords:** piezo–electromagnetic generators, magnetic repulsion, supercapacitors buffer, energy harvesting, electronic pre-regulator

## Abstract

This paper focuses on hybrid piezo–electromagnetic generators, which are assembled from a magnetic repulsion pad made of two disk magnets, a sliding cylindrical magnet placed inside a tube, a coil, and an assembly of piezoelectric elements connected with the magnetic pad, as well as an electronic system for rectification and voltage adjustment. Four piezo–electromagnetic generators have been developed. Two linear generators without magnetic cores were tested and optimized for low-frequency (0.2 Hz…5 Hz) and low-amplitude body movements. The other two generators were also designed to handle high-vibration amplitudes, to generate up to 2.2–2.5 W of power. An algorithm for the calculation and modeling of these hybrid generators is presented, as well as simulation models. In addition, an electronic hybrid voltage converter was realized. It was observed that the system harvesting efficiency was increased by adding a large capacitive buffer made of electrolytic capacitors after the Schottky diode rectifiers bridges. This capacitive buffer, together with the electronic pre-regulator, has the role of limiting the voltage to the desired input value and of being the first charging stage. Finally, in the second charging stage, an electronic converter is used to charge the supercapacitors.

## 1. Introduction

There has long been interest in harvesting energy from human body movements such as walking, step impacts, running, and joint stretching. In this work, we present an approach to efficiently capture energy from a wide range of vibration amplitudes, ranging from low-amplitude motions, like walking, to high-amplitude activities, such as running or jumping, without compromising user comfort or interfering with natural movements. To achieve this, a hybrid piezo–electromagnetic generator was developed, capable of adapting to different vibration intensities while maintaining a minimal physical impact on the wearer. The paper [1] presented an energy-harvesting device designed to capture energy from ultralow-frequency human body movements. This dual piezoelectric and electromagnetic coupling energy harvester converted the swinging motion of human arms into usable power. The device consisted of two piezoelectric transducers Pb(Zr1-xTix)O3 (PZTs), a pair of arc-shaped drive magnets, and two sets of coils. Notably, the coil with the highest number of turns (1000 turns) generated approximately 11 mW of power from arm-swinging movements at an ultralow frequency of 2 Hz. Meanwhile, the piezoelectric system alone, with a peak power output of 0.306 mW, proved inefficient as a standalone solution. The electromagnetic generator significantly enhanced the overall power output, producing over 23 times more energy, with a maximum of 10.65 mW. The study focused on the effect of the cylindrical magnet’s diameter, demonstrating that a larger diameter increased both the open-circuit voltage of the PZT and the induced electromotive force (EMF). However, if there are no geometrical constraints, the magnet’s length is also a key factor in optimizing coil design. This paper [2] investigated a nonlinear energy harvester using magnetic interactions to achieve bistability and broaden the frequency response via the potential well escape phenomenon. The design features a center magnet oscillating within a tube, repelled by top and bottom magnets fixed to caps, as well as four symmetrically placed side magnets. The coil has a 15.9 mm inner radius, 50.8 mm length, and 2000 turns; the center magnet measures 19.05 mm in diameter and length. Experiments near the 5.12 Hz natural frequency produced 100–200 mW of power.

The paper [3] described a multi-mechanism energy-harvesting device combining two electromagnetic generators (EMGs), two piezoelectric generators (PEGs), and one triboelectric nanogenerator (TENG). The EMG unit features a levitated NdFeB permanent magnet (30 mm in diameter, 5 mm in height) positioned within three cylindrical magnets arranged in a triangle. This configuration allows the magnet to vibrate vertically in response to external vibrations, capturing energy without mechanical contact, thus reducing structural fatigue. The coil, wound with 70 μm (too low for consistent outputs) of copper wire, contains approximately 4000 turns for optimal magnetic flux generation. The PEG unit uses PZT ceramic sheets, fixed on conical plates. The levitated magnet’s oscillations impact the PZT sheets, generating an electric current through the piezoelectric effect. The TENG incorporates silicone with carbon nanotubes (CNTs) to improve dielectric properties. The device showed an increasing output with the excitation frequency, peaking at 20 Hz. The PEGs generated a maximum open-circuit voltage of 60 V and 2.8 mA of the short-circuit current, while the TENG produced 78.4 μW at this frequency. The EMGs delivered 36 mW and 38.4 mW, respectively, suggesting that further optimization of the coil and magnet geometry was needed to maximize energy efficiency. At 20 Hz, the PEGs delivered peak outputs of 122 mW. The invention [4] is similar to the one discussed in the present paper but differs in coil configuration, and no piezoelectric stacks are attached at both ends. Konotchick’s design uses two short coils, whereas the present paper employs a single coil of at least twice the magnet’s length. Conversely, for coils exceeding 10,000 turns, the present paper recommends using thicker wire (0.16–0.2 mm) along with a longer magnet and coil to maintain optimal internal resistance and maximize power output. A similar electromagnetic harvester structure is described in [5]. The proposed hybrid harvester comprises four magnets and two copper coils arranged symmetrically along its axis. Two fixed magnets and two attached to a central double circular cone form a sandwich-like suspension structure. Surrounding this structure are three push rods and piezoelectric plates (PZT-5H on Fe-Ni42 alloy), spaced at 120°, converting vertical magnetic oscillations into horizontal displacement. This coordinated dual-axis motion drives both piezoelectric and electromagnetic transducers, yielding a peak output of 80.10 mW at 2 g with a 1 kΩ load. In contrast, a device from [6] uses a magnetoelectric laminate (PZT bonded to Terfenol-D) and three ring magnets to levitate a central magnet. Despite achieving 105 V, it produces only 1.1 mW; even so, the overall efficiency of the hybrid design is increased.

Electromagnetic double-coil structures have been explored in [7,8]. In [7], the harvester increases power delivery to the optimal load across various vibration frequencies, with the most significant gain occurring below its resonance frequency. It also utilizes electrical load matching as a tuning technique to enhance system efficiency. In [8], a method of controlling the distance between non-levitating magnets boosts energy performance, with the harvester’s length-dependent behavior suggesting potential for automatic tuning based on the excitation frequency and the magnitude of mechanical excitation. The review article [9] describes several types of energy harvesters, including ferrofluid-based harvesters, a frequency-tunable piezoelectric harvester with geometric self-adjustment, a frequency-tunable piezoelectric cantilever energy converter with preload, and frequency-tunable PVDF extensional mode resonators with a centered seismic mass. It also covers multifrequency cantilever arrays and harvesters that contain free-moving spherical inertial masses (either magnetic or non-magnetic) inside circular cavities or cylindrical tubes with double-coil electromagnetic structures. Another design presented in [10] features a rigid magnet ball freely moving inside a copper coil-wrapped box, with piezoelectric diaphragms placed at the top and bottom. This 30 cm^3^ converter generates 5 V peak voltages from the piezoelectric diaphragms and 200 mV alternating voltages from the copper coils under 0.1 g of acceleration and a 10 Hz frequency. The following hybrid generator [11] integrated three units—EMG (electromagnetic generator), PEG (piezoelectric generator), and TENG (triboelectric generator)—to deploy synergistic outputs. The EMG unit produced current through magnetic flux changes in a Cu-centered coil, based on Faraday’s law. The PEG unit generated a piezoelectric current when the magnet impacted the PVDF films at both ends of the device. Simultaneously, the TENG unit generated current due to the triboelectric charges on the magnet’s surface, creating a potential difference between the two electrodes. All three units, triggered by the magnet’s movement, produced synchronized outputs. The hybrid generator achieved a maximum output of 63 µW at 140 MΩ for the TENG unit, 87.1 mW at 500 Ω for the EMG unit, and 26.17 mW at 70 kΩ for the PEG unit. Various types of hybrid piezoelectric cantilever structures, with magnets attached to the ends and coils mounted near the magnets, are presented in [12,13,14,15,16]. These structures generate only several milliwatts of power, with peak powers ranging from 3 mW to 11 mW. The bridge-type mechanism [17], featuring a piezoelectric stack at the center, provides only 2.66 µW due to the high rigidity of the entire system.

This study aims to maximize the power output from human body movements using a hybrid piezo–electromagnetic generator. To achieve this, the number of coil turns was increased to be between 9000 and 12,500, while minimizing the internal resistance of the coil [18]. The maximum voltage output was set, based on the design, to 40 V to avoid damaging the electronics and electrolytic capacitors, which are rated for 50 V. This was achieved theoretically and experimentally verified by using magnets with a diameter between 10 and 15 mm and a corresponding length of 20 to 40 mm. Also, the optimal coil geometry was designed with a length just over twice the magnet length, providing adequate space for windings, while ensuring the coil’s mean radius did not exceed the magnet diameter. All previously mentioned theoretical aspects are presented in Section 2. The device components and all design considerations are described in Section 2.

During the optimization process, four electromagnetic generators were constructed and are presented in Section 3. Two of these featured end caps with piezoelectric PZT-type membranes and repelling magnets attached at the center to capture energy from both body vibrations and the magnetic cushion. The magnetic cushion consists of a central magnet that levitates between two repelling magnets, minimizing friction between mechanical components. This design increases the oscillating frequency and damping time by a factor of four to six, compared to the body’s natural movement frequency.

In Section 3, the Results Section, experiments performed with all generators are presented, and the damping oscillation of the center magnet inside the magnetic cushion was caught by using an oscilloscope and by monitoring the coil voltage for a human walking at a frequency of 1.5–1.8 Hz and for a human running or jumping at a frequency of 3–4 Hz.

In the final part of this paper, the roles of the large capacitive buffer and the pre-regulator are discussed in the context of improving harvesting efficiency. To serve this purpose, a switching pre-regulator, aided by an energy flow management module, was used to maintain the voltage within a range of 6 to 12 V for the second charging stage. This stage included an additional electronic converter, a 5 V switching regulator, responsible for charging the supercapacitors. The effective design of the switching pre-regulator, the energy flow management unit, and the final switching electronic converter enables a power efficiency exceeding 80%.

## 2. Materials and Methods

### 2.1. Design of Hybrid Piezo–Electromagnetic Generator

The hybrid piezo–electromagnetic generator is a type of linear and non-resonant generator operating in oscillating mode, under the vibrational forces of a moving magnet inside a pipe. The hybrid piezo–electromagnetic generator, according to patent application No. A/00997 dated from 29 November 2018 [18], consists of three magnets placed in a magnetic repulsion cushion system (1), two magnets (1b) (and/or two springs (7)) being arranged at one end and the other of the pipe (5) inside which the central magnet (1a) oscillates and levitates, inducing an EMF in coil (2) from the body movement. The central multiturn coil (2) has a large number of turns, higher than 5000 and limited to 12,500, in order to not exceed the induced electromotive voltage of 45 V. The turns are placed in several rows and centrally on the pipe (5), and the coil thickness should not exceed 6 mm to maximize the flux linkage. The central coil should have a minimum length of at least twice the length of the sliding magnet. In addition, it is necessary to leave equal free spaces at the ends of the pipe (5) of at least the length of the sliding magnet, plus the magnets (1b) length and a 5 mm reserve for the magnetic repulsion. The magnets (1b) at the ends of the pipe are each glued to an assembly with one or more piezoceramic elements and brass disks arranged mostly in parallel connection because the maximum rectification voltage exceeds 31 V for a single 40 mm brass disk and a 24 mm piezoceramic element. This voltage peak is directly linked to the magnetic repulsion force of 10–60 N and depends on the human body activity of running or jumping. The assembly of piezoelectric elements (3) with the brass disks (9) that are glued at the ends (16), on the circumference, to two rigid caps (4), oscillates simultaneously with the magnetic repulsion system and the central magnet. The assembly also has ring-type rubber (or plastic) insulators and spacers (8) for electrical and mechanical shock protection.

Inside the device’s outer tube (6), there is a ring-type voltage rectification and regulation system of electrolytic capacitors (11). These rings are also protected by the same spacers (8). The inner diameter of the ring corresponds to the outer diameter of the sliding tube (5). A sliding tube with a maximum thickness of 1 mm was used to avoid very high magnetic field dispersion losses.

There are also the ventilation holes (14), provided for magnet diameters greater than 10 mm, in order to not slow down the movement of the sliding magnet in contact with the air inside the pipe (5). In his patent, John A. Konotchick also refers to ventilation holes. The inventor filed and received the US Patent No. 5,818,132 in 1998 [4] for a linear motion electric power generator optimized for low-power mechanical forces and vibrations. The design accommodates various nonferrous tube materials, with thin-walled brass being the preferred choice. The core component, the center magnet, was a rod magnet (Alnico or NdFeB), measuring 12.7 mm in diameter and 19 mm in length. The device features two coils, each 19 mm long, wound with either 8000 turns of 0.16 mm in diameter wire or up to 10,000 turns of 0.1 mm–0.16 mm in diameter transformer wire. These coils are bound with acrylic cement and positioned on the tube with a 19 mm separation. The end magnets are 25.4 mm in diameter, with 6.35 mm thick ceramic disks, arranged in polar opposition to the center magnet, suspending it via repulsive magnetic force. Although the number of turns matches those presented in this earlier patent, optimal internal resistances were not ensured, as in the present invention.

Unlike previously reported systems (in the introduction), which include ultralow-frequency arm-swing harvesters generating only milliwatts of power, bistable magnet systems limited by narrow frequency bandwidths, complex multimodal triboelectric–piezo–electromagnetic hybrids prone to structural fatigue, and designs with either poor magnetic optimization or no piezoelectric integration, the present work introduces a compact, non-resonant, hybrid piezo–electromagnetic generator. It features a magnetically cushioned oscillating core and end-capped piezoelectric elements. This configuration enables prolonged, amplified oscillations, up to six times the body motion frequency, and achieves superior energy conversion efficiency, reaching up to 2.5 W. The generator adapts to low-frequency motion, ranging from 0.2 to 5 Hz, due to a high number of coil turns. It also exhibits robust modularity. Additionally, a custom energy management circuit is incorporated, which includes capacitive buffering and pre-regulated charging stages. These features represent a significant advancement in wearable energy harvesting through combined mechanical and electrical design optimization, practical implementation, and scalability. Regarding scalability and modular design, the harvester can be scaled up by adding more piezoelectric elements to the stack actuator, enabling up to 0.3 W of piezoelectric output using 16 elements (8 per end cap). The electromagnetic output can also be doubled to around 5 W by increasing the winding diameter and optimizing coil turns and dimensions, while preserving the already-optimized length-to-diameter ratio. Furthermore, a modular setup allows multiple units to be combined in a single enclosure, potentially delivering a total output of up to 10 W.

**Figure 1:**
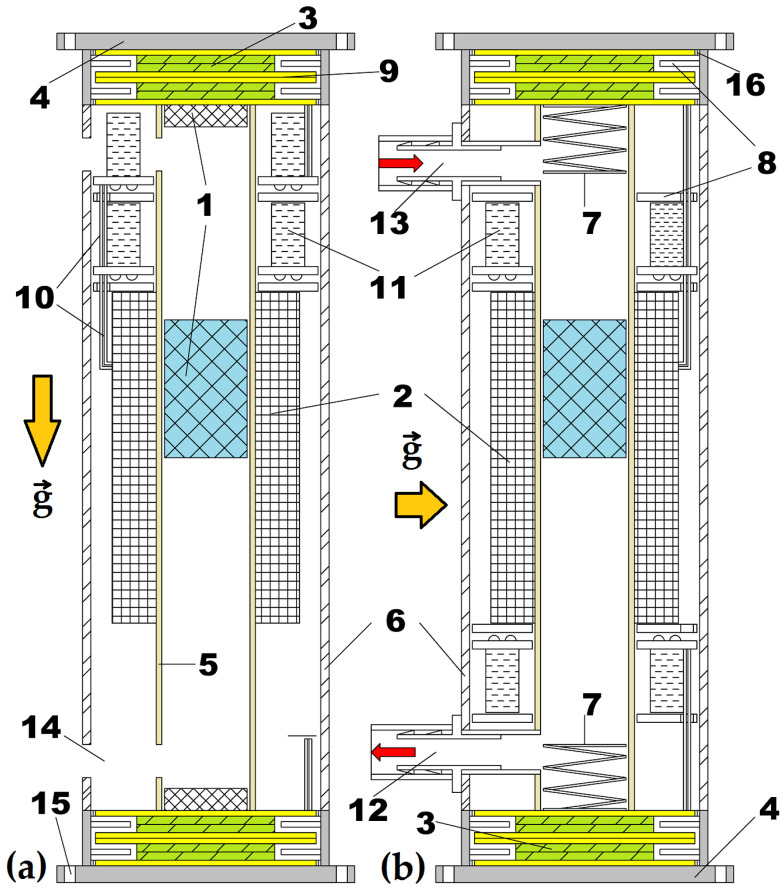
(**a**) Piezo–electromagnetic generator design for energy harvesting of human body motions; (**b**) Piezo–electromagnetic generator design for energy harvesting of human body breathing.

A key advantage of the proposed piezo–electromagnetic generator over existing devices is its adaptability to two distinct scenarios, one of which is rarely addressed in the literature. In the first scenario, the generator harvests energy from vibrations produced by the legs, arms, and torso. As illustrated in Figure 1a, the direction of gravitational acceleration is indicated by $\underset{g}{\to }$, and the device is secured around the torso using a belt. In the second scenario, the generator utilizes breathing-induced motion to accumulate energy, a case that has received limited attention in prior studies. As shown in Figure 1b, nozzles (13) and (12) are integrated into openings (14) to enable this functionality, with the gravitational acceleration direction again represented by $\underset{g}{\to }$.

The hybrid generators are capable of harvesting energy from human breathing using a two-way valve system. One valve opens during exhalation, allowing airflow to activate the generator, while the other remains closed. Both valves are positioned at opposite ends of the generator to control the direction of airflow throughout the breathing cycle. During inhalation, the first valve closes, and the second valve opens again during the next exhalation cycle, enabling continuous energy capture from each breathing phase.

An alternative and ingenious design involves the use of reed valves, a type of two-way valve that already exists (on the market). Reed valves operate by responding to pressure gradients, opening when the pressure difference across the valve lifts the flexible reed element. Additionally, reed switches, which are passive magnetic switches, can be integrated to detect the position of the center magnet and actively control the valve, enabling it to open precisely when needed based on the magnet’s location.

In short, the air exhaled through the exhalation passes through the hose connected to the nozzle (13), enters the inside of the pipe (5), and pushes the magnet with pressure. The same process is also repeated for breathing, but the present paper will not focus on this aspect. The outer covers (4) are fixed by means of threaded rods (two or three in number) and fastening screws (not specified in the drawing) to the entire body of the protective pipe (6) and the inner sliding pipe (5), with both pipes being mechanically processed to the same length.

This piezo–electromagnetic generator can be equipped with two repulsion springs (7), but the magnetic cushion (1) will be much more efficient. The magnetic cushion is able to retain and increase the damped oscillation duration, with the body movement frequency being amplified four times.

The hybrid piezo–electromagnetic generator, according to this patent application, is obtained by assembling the main components, which are the magnetic repulsion cushion, the sliding magnet, and the central coil as well as the ensemble of the piezoelectric elements mechanically connected to the magnetic cushion. The electronic rectification system, electrolytic capacitors buffer, and voltage regulation system are mounted inside and partially outside the protective pipe. These hybrid piezo–electromagnetic generators, without a magnetic core, are optimized for low-frequency and low-amplitude body movements, by increasing the number of turns and choosing an optimal conductor of 0.12…0.25 mm, so that the internal resistance of the coil is not very high. They have an additional magnetic cushion to obtain a slowly damped mechanical oscillation and an amplified vibration rate and to protect the sliding magnet from mechanical shocks. Each magnetic cushion is mechanically attached to the piezoceramic elements (PZT-PbZrTiO3 type) and brass disks, which help to supplement the electrical energy requirement.

To reduce air damping, one of the following modifications can be used [4]: adding notches at the tube ends, drilling holes along the tube, ensuring sufficient tube diameter clearance for airflow around the magnet, or using toroidal magnets with holes. Here, two 6 mm holes were drilled at both inner tube ends to reduce air damping or to attach hoses and nozzles for harvesting breathing energy. In this case, no magnetic nanofluid can be added inside the device, between the tube and the oscillating magnet, to reduce the friction [19]. Another option will be to use toroidal magnets with holes to reduce air damping and to seal the tube when adding the magnetic nanofluid inside. Another problem is that piezoceramic materials are prone to fatigue and failure under dynamic bending stress. Since the bending beam structures alone lack sufficient reliability and durability, an alternative approach is to attach a magnet to a doubly clamped elastic rod subjected to nonlinear vibrations. This bow-shaped or cymbal structure enhances excitations, leading to large compressive loads in piezoelectric materials and proportional voltages, between 32 V and 109 V, as the vibration frequency increases from 1 Hz to 15 Hz [20,21,22].

For energy harvesting from the human body, three types of permanent magnet topologies can be utilized [23]: linear generators (resonant or non-resonant) operating in oscillating mode under vibrational forces, rotational generators driven by a steady torque, and hybrid generators with unbalanced rotors that convert linear motion into rotational motion. Unbalanced micro-rotors have been used since 1954 in automatic watch movements, where hand and body movements rewind the mainspring. Based on this principle, a flat stator with spiral windings and an unbalanced rotor with permanent magnets can be designed for energy-harvesting applications.

The present paper focuses on a non-resonant linear generator operating in oscillating mode under the vibrational forces of footsteps. For this application, it is preferable to attach the device to the waist (torso) using a belt. The electromagnetic generator designs discussed in this section are based on the experimental data and geometries of the PEG II and EG I prototypes, as presented in the Results Section. These generators incorporate 9220 and 12,500 coil turns, respectively. The oscillation frequency of the magnet was determined experimentally from the timing of the induced voltage peaks, yielding approximately 25 Hz during running and 8 Hz during walking. Given the complex dynamics involved, it is challenging to accurately predict magnet behavior through theoretical modeling alone. Additionally, the walking step frequency, ranging from 1.5 to 1.8 Hz, was measured from experimental waveforms by identifying the corresponding voltage peaks.

The electromagnetic generator design was based on three theoretical models. Firstly, the simplest model that was analyzed was the magnetic circuit model. The flux linkage between the magnet and coil was the calculated function of the air gap distance between the leading magnet end and the coil’s center. The second model was a 2D analytical model based on magnetic field variation with an $x_{c}$ air gap distance. The third model was based on the same magnetic field variation, but here the flux linkage between coil and magnet depended on the relative position of the magnet center from the coil’s center.

**Figure 2:**
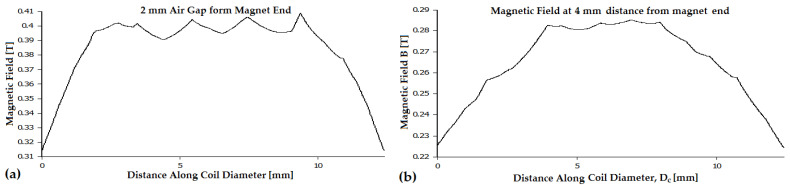
(**a**) Magnetic field of a 10 mm in diameter, 20 mm long magnet along the coil diameter, at a 2 mm distance from the magnet leading end (FEMM simulation); (**b**) magnetic field of a 10 mm in diameter, 20 mm long magnet along the coil diameter, at a 4 mm distance from the magnet leading end (FEMM simulation).

The magnetic field of a 10 mm diameter, 20 mm long magnet with N48 magnetization (residual magnetic field $B_{r}$ of 1.4 T) was analyzed at various distances from the magnet’s leading edge, ranging from 0.1 mm to 18 mm (see Figure 2a,b).

**Figure 3:**
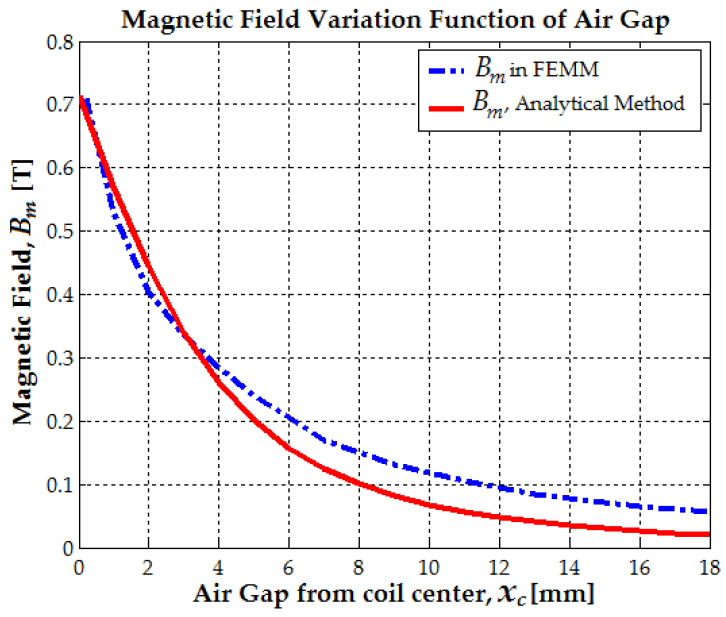
Magnetic field variation function of the air gap distance from the magnet leading end.

These distances can be considered as the air gap $x_{c}$, measured from the magnet’s end to the center of the coil. The magnetic field exceeds 0.9 T only near the corners of the magnet, and the average magnetic field at a 0.1 mm gap does not exceed 0.75 T. At a 0.2 mm air gap, the average magnetic field along the coil’s 11.4 mm diameter is 0.71 T (see Figure 3). The average magnetic field at a 0.5 mm air gap, along both the magnet and coil diameter, is 0.6 T. The magnetic field simulation of a 10 mm diameter, 20 mm long magnet placed inside the coil was performed using Dirichlet boundary conditions. To ensure precise results, seven boundary layers were applied.

Subsequently, the magnetic field density was analyzed in 1 mm steps, from 1 mm to 18 mm. At a 1 mm air gap, the average magnetic field, extracted from 2D FEMM simulations, was 0.53 T. The magnetic field density was then analyzed at increasing air gaps, starting from 2 mm. At a 2 mm air gap, the average magnetic field along the coil’s diameter dropped to 0.40 T. At a 3 mm air gap, the average magnetic field was 0.335 T. As the air gap increased from 4 mm to 8 mm, the average magnetic field decreased from 0.285 T to 0.15 T (see Figure 3). With further separation, at a 10 mm air gap, the magnetic field density reached 0.11 T. Beyond 15 mm, the magnetic field became too weak to induce significant electromotive voltage, reaching only 0.07 T.

The total magnetic flux linkage through a coil due to a cylindrical magnet depends on how the magnetic field $B_{m}$ varies along the coil’s axis. The general expression for the axial field of a uniformly magnetized cylinder along its central axis is [24].

(1) $$B_{m}=\int_{-kl_{m}}^{+kl_{m}}\frac{B_{r}}{2}\frac{r_{m}^{2}dx}{{\left[{\left(x_{c}-x\right)}^{2}+r_{m}^{2}\right]}^{3/2}}=\frac{B_{r}}{2}\left(\frac{x_{c}+kl_{m}}{\sqrt{r_{m}^{2}+{\left(x_{c}+kl_{m}\right)}^{2}}}-\frac{x_{c}-kl_{m}}{\sqrt{r_{m}^{2}+{\left(x_{c}-kl_{m}\right)}^{2}}}\right)$$

Here, when k=1/2, the general expression for the magnetic field is considered, with the magnet center as a reference. $B_{r}$ is the residual magnetic flux density or remanence of the magnet, $x_{c}$ is the position along the coil axis or the air gap, $r_{m}$ is the radius of the magnet, and $l_{m}$ is the length of the magnet (assuming a symmetric cylindrical magnet).

This formula was derived from the integral of the Biot–Savart law for a uniformly magnetized cylinder along its axis. A similar analogy between the magnetic field produced by a cylindrical permanent magnet and a cylindrical coil or solenoid and the respective analytical equations were presented in [24]. Here, an experimental setup was used for measuring the static and dynamic magnetic fields with the distance variation between 2 mm and 100 mm. The values obtained from the numerical models were compared with the ones obtained from the experimental setup, and the data fit quite well. In the interval 0–80 mm, the relative errors between the numerical model data and experimental data had values below 5%. This means that 2D static magnetic field simulations made in FEMM 4.2 can also be validated for this case.

**Figure 4:**
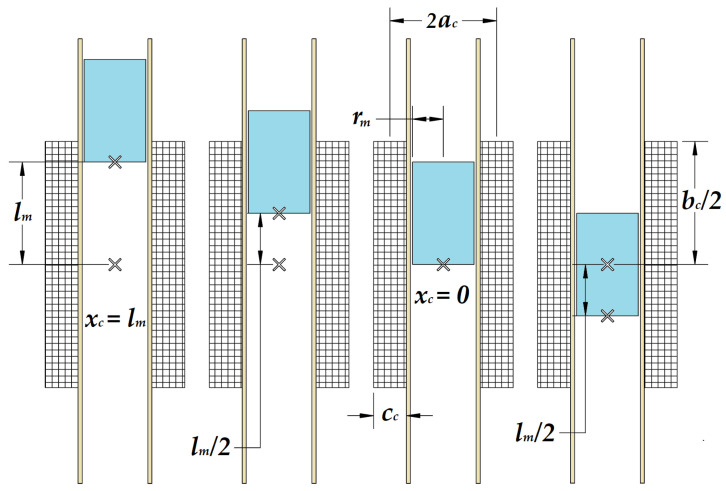
Diagram of a moving magnet from the coil edge to the geometric center, with the air gap defined as the distance between the leading edge of the magnet and the coil’s center.

The movement of the magnet is defined as traveling along its axis from one end of the coil toward the coil’s center, and this is the case described in Figure 4. The reference point for measuring the magnetic field and magnetic flux is the leading end of the magnet—the end that is moving toward the coil’s center. The air gap is defined as the distance between this magnet end and the geometric center of the coil. With this reference, the previous Formula (1) is modified to (2), with the mention that k=1/2.

(2) $$B_{m}\left(x_{c}\right)=\frac{B_{r}}{2}\left(\frac{1+\frac{x_{c}}{l_{m}}}{\sqrt{{\left(\frac{r_{m}}{l_{m}}\right)}^{2}+{\left(1+\frac{x_{c}}{l_{m}}\right)}^{2}}}-\frac{\frac{x_{c}}{l_{m}}}{\sqrt{{\left(\frac{r_{m}}{l_{m}}\right)}^{2}+{\left(\frac{x_{c}}{l_{m}}\right)}^{2}}}\right)$$

When the cylindrical magnet begins to descend, it starts well above the coil’s entrance. At this stage, if the magnet is more than half its length away from the coil, there is no significant magnetic flux threading through it, and consequently, no electromotive force (EMF) is induced. As the magnet approaches the coil’s entrance, some of its magnetic field begins to thread through the top turns of the coil. Since the magnetic flux is now changing over time, an EMF is induced, marking the initial rise in the EMF voltage graph. As the magnet continues its descent and enters the coil, an increasing portion of its magnetic field threads through the coil’s turns. The faster the total flux changes, the greater the induced EMF. The EMF reaches its peak when half of the coil’s turns are threaded by the flux—this occurs when the magnet position is changing between half and fully inside the first half of the coil, with one end near the coil’s center. At this point, the air gap between the magnet end and the coil center is minimal, maximizing the flux linkage. The magnetic field lines above and below the magnet point in the same direction. When the magnet’s center aligns with the middle of the coil, the flux increase in the upper half is precisely canceled by the flux decrease in the lower half. As a result, the net change in flux is zero, causing the EMF to drop to zero. This corresponds to the midpoint of the EMF voltage graph, where the curve crosses the horizontal axis.

As the magnet continues to fall beyond the midpoint, the EMF begins to decrease, representing its movement toward the lower half of the coil. When the magnet end exits, more flux leaves than enters, causing the EMF to reverse polarity. This results in a negative voltage section in the EMF vs. time graph, mirroring the earlier positive section. Mathematically, the magnetic flux is the integral of the magnetic field over an area, and its time derivative determines the induced EMF. By Stokes’s theorem, the area integral of the curl of a vector field is equivalent to the line integral of the field along the boundary. In this case, the line integral of the electric field corresponds to the induced EMF.

The magnetic circuit of a magnet oscillating through a coil can be expressed as a function of air gap permeance and magnet permeance. Permeance of the air gap $x_{c}$ can be written as $P_{x_{c}}=\mu_{0}S_{c}/x_{c}$, and the magnet permeance is $P_{m}=\mu_{0}{\mu_{r}S}_{m}/l_{m}$. The magnetic flux passing through the coil is $\Phi_{c}$ and the magnetic flux of the magnet is $\Phi_{m}$.

(3) $$\frac{d\Phi_{c}}{dx_{c}}=\frac{d}{dx_{c}}\left(\frac{\Phi_{m}P_{x_{c}}}{P_{x_{c}}+P_{m}}\right)=B_{r}S_{m}\frac{S_{c}}{{\left(S_{c}+\frac{\mu_{r}S_{m}x_{c}}{l_{m}}\right)}^{2}}\left(\frac{{-\mu }_{r}S_{m}}{l_{m}}\right)$$

When the magnet is oscillating back and forth along the coil axis due to the magnetic cushion, the air gap is also changing. The air gap is the expressed function of the distance from the magnet’s leading end and coil’s geometric center (see Figure 4).

(4) $$\frac{d\Phi_{c}}{dt}=\frac{d\Phi_{c}}{dx_{c}}\frac{dx_{c}}{dt}=-\frac{d\Phi_{c}}{dx_{c}}x_{cmax}\sin\left(\omega_{m}t\right),\;x_{c}=x_{cmax}\cos\left(\omega_{m}t\right)\;,$$

(5) $$U_{c}={-N}_{c}\frac{∆\Phi_{cmax}}{∆t}={-N}_{c}\frac{∆\Phi_{cmax}}{∆x_{c}}\frac{∆x_{c}}{∆t}.$$

When the air gap is at its minimum, $x_{c}=0$, the reference end of the magnet is exactly at the coil’s center. Since the coil is twice as long as the magnet, the entire magnet is fully enclosed within the coil at this position. The rate of change in flux is the highest, generating the maximum electromotive force (EMF). This corresponds to the behavior of the magnetic circuit described by Formula (3). The EMF peak occurs when the derivative of the magnetic flux is at its maximum, which happens when the magnet’s end is near the center of the coil but not fully inside.

When the air gap is at its maximum, $x_{c}=l_{m}$, the reference end of the magnet is positioned one magnet length away from the coil center, meaning that the entire magnet is just outside the coil. In this position, there is minimal flux linkage, resulting in the minimum induced EMF.

(6) $$∆\Phi_{m}=∆B_{m}S_{c}$$

In a linear oscillatory movement, maximizing the magnetic field variation $∆B_{m}$ with the air gap is more important than simply increasing the magnet’s diameter when optimizing the magnetic flux through the coil constant inner surface $S_{c}=\pi r_{ic}^{2}$. While the magnet’s diameter remains important, as it directly determines the area, the magnet’s length has a greater impact on the generated power.

The maximum possible magnetic field variation is given by $∆B_{m}=B_{2m}-B_{1m},\;B_{1m}=B_{m}\left(x_{c}\right)$. Here, $B_{2m}$ represents the magnetic field at a distance $l_{m}$ from the coil center to the magnet’s leading end, corresponding to the moment the magnet is just exiting the coil. In contrast, $B_{1m}$ is calculated for $x_{c}=0$, where no air gap exists between the magnet’s leading end and the coil center (see Formulas (7) and (8)). This difference directly corresponds to the flux variation factor, $k_{f}$.

(7) $$B_{2m}\left(l_{m}\right)=\frac{B_{r}}{2}\left(\frac{2}{\sqrt{{\left(\frac{r_{m}}{l_{m}}\right)}^{2}+4}}-\frac{1}{\sqrt{{\left(\frac{r_{m}}{l_{m}}\right)}^{2}+1}}\right)$$

(8) $$k_{f}=\frac{2}{\sqrt{{\left(\frac{r_{m}}{l_{m}}\right)}^{2}+4}}$$

The normalized flux variation factor, $k_{f}$, approaches 1 (0.97) when the magnet radius, $r_{m}$, is half of its length, $l_{m}$. Since the EMF increases with $k_{f}$, a magnet with a radius larger than its length will result in $k_{f}<0.89$, reducing the peak EMF voltage and generated power.

(9) $$U_{c}\left(x_{c}\right)={\omega_{m}N}_{c}S_{c}∆B_{m}\left(x_{c}\right)\frac{x_{cmax}}{l_{m}}$$

Formulas (10) and (11) give the calculation of magnetic flux derivative and EMF expression for an oscillatory movement of the magnet. The oscillatory linear motion of the magnet depends on both the air gap and time. While the magnetic flux expression depends only on changes in the air gap and not necessarily on time, the EMF amplitude will be at its maximum when the air gap variation is greatest (between 0 and $l_{m}$) and when the mechanical oscillation frequency $\omega_{m}$ is at its maximum. The magnetic cushion formed by repelling magnets at both pipe ends has the role of shifting the oscillating frequency of the body movement to a higher value, between three to four times more. In addition, if the magnet is short, a lower air gap variation will be obtained.

(10) $$∆\Phi_{cmax}=S_{c}\frac{dB_{m}}{dx_{c}}=\left(\frac{B_{r}S_{c}}{2}\right)\frac{r_{m}^{2}}{{\left[r_{m}^{2}+{\left(x_{c}+kl_{m}\right)}^{2}\right]}^{3/2}}$$

(11) $$U_{e}\left(t\right)={\omega_{m}N}_{c}\left(\frac{B_{r}S_{c}}{2}\right)\;\frac{x_{cmax}r_{m}^{2}}{{\left[r_{m}^{2}+{\left(x_{c}+kl_{m}\right)}^{2}\right]}^{3/2}}\sin\left(\omega_{m}t\right)$$

The EMF reaches its peak when the magnet is positioned so that half of the coil’s turns are threaded by the flux, resulting in the maximum rate of change in magnetic flux. Since the coil is twice the magnet’s length, the peak EMF typically occurs when the magnet is near the center of the coil but not fully inside, where the flux linkage changes most rapidly. In this case, k=1/2 in Formula (12).

(12) $$U_{c}\left(x_{c}\right)=\left(\frac{{{\omega_{m}N}_{c}B}_{r}S_{c}}{2}\right)\;\frac{x_{cmax}r_{m}^{2}}{{{\left(kl_{m}\right)}^{3}\left[\frac{r_{m}^{2}}{{\left(kl_{m}\right)}^{2}}+{\left(1+\frac{x_{c}}{kl_{m}}\right)}^{2}\right]}^{3/2}}$$

**Figure 5:**
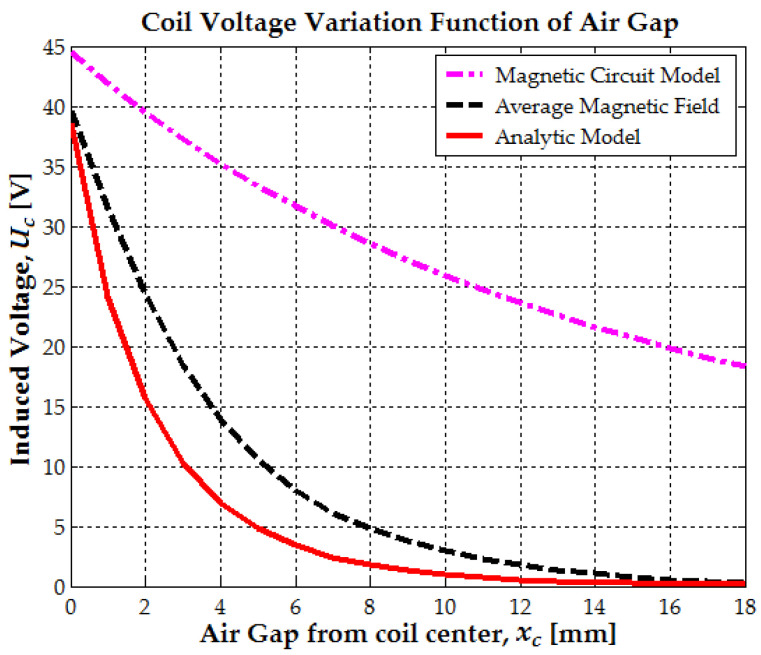
Induced electromotive force (EMF) of a 10 mm diameter, 20 mm length magnet with the leading edge moving from the center to the end of the coil as a function of the air gap between the magnet and the coil’s center.

In Figure 5, all previous mathematical models are compared, as the air gap $x_{c}$ varies between 0 and $l_{m}$ or $\left(b_{c}+l_{m}\right)/4$, where $b_{c}$ represents the coil length. The results indicate that the magnetic circuit model can reasonably estimate only the maximum flux variation and the maximum induced voltage.

The average magnetic field, determined using Formulas (6), (7) and (2) as a function of the air gap $x_{c}$, demonstrates good accuracy, with relative errors below 5% [24]. The maximum induced voltage when the magnet’s leading end approaches the coil center ($x_{c}=0$) can be expressed in terms of the flux variation factor as follows: $U_{cmax}\left(0\right)={\omega_{m}N}_{c}S_{c}\left(\frac{B_{r}}{2}\right)k_{f}$. This is referred to as the average magnetic field because, as shown in Figure 2b, a constant magnetic field value must be selected across the entire coil diameter to be used further. A limitation of the analytical model is its inability to estimate the magnetic field along the coil radius. However, it is certain that as the coil radius increases, the magnetic field decreases, eventually reaching a point where no significant flux variation is observed at the coil’s ends. If the coil has a large radius relative to the magnet, the outermost turns will see minimal flux change, leading to a reduced or negligible EMF.

**Figure 6:**
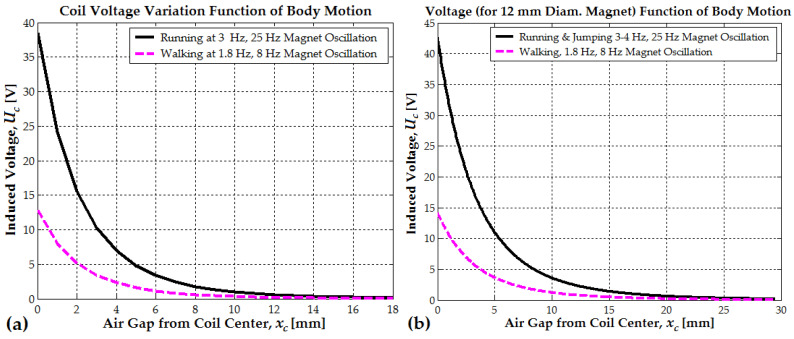
(**a**) Induced EMF of a 10 mm × 20 mm magnet vs. air gap from the coil center to the magnet leading edge. (**b**) Induced EMF of a 12 mm × 36 mm magnet vs. air gap from the coil center to the magnet leading end.

For optimal efficiency, the mean coil radius $a_{c}$ should be smaller than the magnet diameter ($2r_{m}$). At a distance of 3.5 mm from the magnet’s edge, the magnetic field strength is approximately 0.25 T. The induced electromotive force (EMF) not only depends on the air gap variation but also on the oscillation frequency $\omega_{m}$. The mechanical oscillation frequency of the magnet varies with the type of body movement, as shown in Figure 6a,b. When walking, the maximum induced EMF per step is 12 V, while when running, it increases to 38 V. This aligns well with the experimental data from Section 3.

These designs were developed based on experimental data and geometric considerations. For the magnet measuring 10 mm × 20 mm, the coil parameters were as follows: a length of 52 mm, a thickness of 6 mm, an outer radius of 12.5 mm, a total number of turns of 9220, and an enameled wire (winding) outer diameter of 0.12 mm. For the 12 mm × 36 mm magnet, the corresponding coil specifications were as follows: a length of 82 mm, a thickness of 5 mm, an outer radius of 13 mm, a total number of turns of 12,300, and a wire outer diameter of 0.15 mm. The outer diameter of the central magnet gliding tube was 13 mm for the 10 mm diameter magnet and 16 mm for the 12 mm diameter magnet. The standard wall thickness of the tube was approximately 1 mm.

### 2.2. Design of Hybrid Generator with Magnet Moving from Center

**Figure 7:**
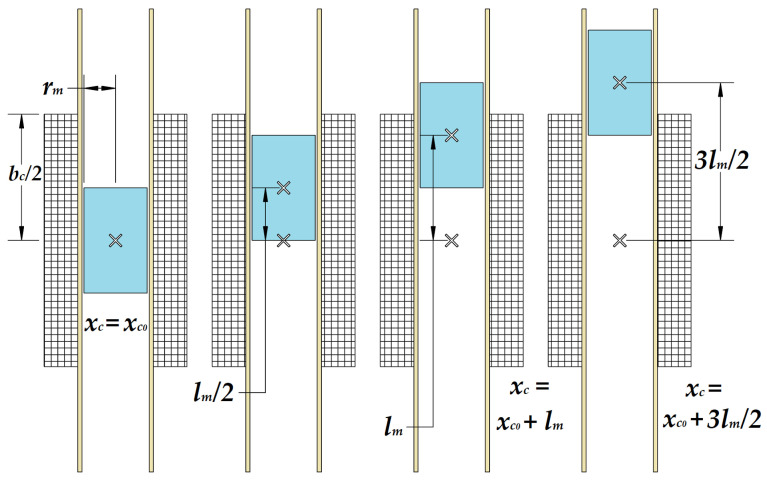
Diagram of a moving magnet from the centered position inside the coil to the edge of the coil; the relative position of the magnet is shifted based on the coil length ($\approx 2l_{m}$).

If the magnet moves from the coil center toward one side, the magnetic flux calculation method differs slightly. In this case, the relative position between the magnet’s center and the coil’s center must be taken into account, considering that the coil length is approximately $2l_{m}$ (see Figure 7).

When the magnet is centered inside the coil, the induced EMF is zero. When the leading end of the magnet moves from position $x_{c}=x_{c_{0}}+l_{m}/2$ and reaches $x_{c}=x_{c_{0}}+l_{m}$, the magnet is half inside and half outside the coil. This position corresponds to the maximum induced EMF. When the magnet center reaches the $3l_{m}/2$ position, the induced EMF is minimal.

Because the coil is twice the magnet’s length, or a little more, the key positions to consider are $x_{c}=x_{c_{0}}$, meaning that the magnet is centered within the coil and ${x_{c}=x}_{c_{0}}+\frac{3l_{m}}{2}$, meaning that the magnet’s leading end reached the coil edge. This position corresponds to a k=2 value. This suggests that the below formula accounts for a relative position shift based on the coil length. Formula (3) is valid because it explicitly considers how the coil length modifies the effective flux linkage between the coil and magnet.

(13) $$x_{c}+\frac{l_{m}}{2}=x_{c_{0}}+\frac{3l_{m}}{2}+\frac{l_{m}}{2}\Rightarrow k=2$$

When k=2, the reference is shifted to point outward from the coil. This shift better captures how the coil interacts with the magnet as it moves outside the coil. This shift considers the total flux linkage across the coil instead of the magnet’s local field (see Formula (15)).

(14) $$U_{c}\left(x_{c_{0}}\right)=\left(\frac{{{\omega_{m}N}_{c}B}_{r}S_{c}}{2}\right)\;\frac{x_{cmax}r_{m}^{2}}{{\left(kl_{m}\right)}^{3}}\left(\frac{1}{{\left[\frac{r_{m}^{2}}{{\left(kl_{m}\right)}^{2}}+{\left(1-\frac{x_{c_{0}}}{kl_{m}}\right)}^{2}\right]}^{3/2}}-\frac{1}{{\left[\frac{r_{m}^{2}}{{\left(kl_{m}\right)}^{2}}+{\left(1+\frac{x_{c_{0}}}{kl_{m}}\right)}^{2}\right]}^{3/2}}\right)$$

(15) $$U_{cmax}\left(l_{m}\right)=\left(\frac{{{\omega_{m}N}_{c}B}_{r}S_{c}}{2}\right)\;{\left(\frac{r_{m}}{l_{m}}\right)}^{2}k_{f};\;k=2;\;k_{f}=\frac{1}{{\left[{\left(\frac{r_{m}}{l_{m}}\right)}^{2}+1\right]}^{3/2}}-\frac{1}{{\left[{\left(\frac{r_{m}}{l_{m}}\right)}^{2}+9\right]}^{3/2}}$$

**Figure 8:**
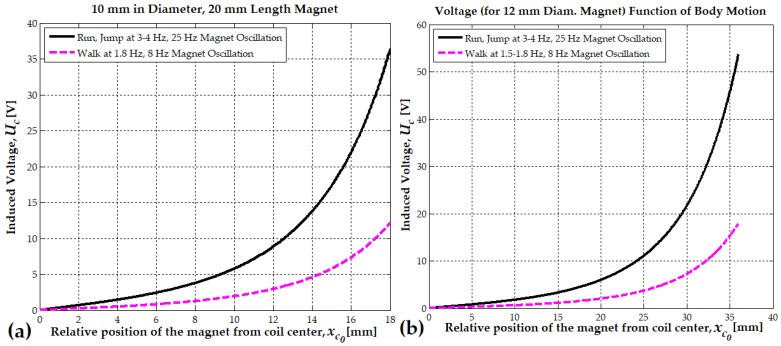
(**a**) Induced EMF of a 10 mm × 20 mm magnet vs. the relative position of the magnet from the coil’s center. (**b**) Induced EMF of a 12 mm × 36 mm magnet vs. the relative position of the magnet from the coil’s center.

Figure 8 illustrates the variation of induced voltage as a function of the magnet’s relative position. When the relative position approaches $l_{m}$, the magnet is half inside and half outside the coil, generating the maximum EMF. For a 10 mm diameter magnet, at a distance of $\left(b_{c}+l_{m}\right)/4=18\;mm$ (with a coil length of 52 mm), the induced voltage is 11.2 V when walking and increases to 36.5 V when running (Figure 8a). These values are lower than those in Figure 6a, indicating that the magnet is not fully outside the coil but remains 2 mm inside. In comparison, for a 12 mm diameter magnet, with a coil length of 82 mm, the corresponding distance is approximately 30 mm, meaning that the magnet remains 6 mm inside the coil. At this position, the induced voltage reaches 14.8 V when walking and 42.5 V when running (Figure 6b). Finally, in Figure 8b, when the magnet is fully outside the coil, the induced EMF increases significantly, reaching 18 V when walking and 54 V when running, values notably higher than the previous case (Figure 6b).

Formula (14) can be generalized for any magnet length. For example, when k=4, the magnet length is one-fourth of the coil length (10 mm). Similarly, when $k=4\left(1/p\right),\;p=1.5$, the magnet length can be considered 15 mm.

Because the oscillating frequency of the human body is low, a maximum of 4 Hz when running, and the flux linkage is in the air, inductive reactance can be neglected in this case, with the total impedance being the inner resistance of the coil:

(16) $$P_{cmax}=\frac{U_{c}^{2}}{R_{c}}=\frac{U_{c}^{2}S_{w}}{\rho 2\pi a_{c}N_{c}},\;S_{w}=\frac{\pi d_{w}^{2}}{4}$$

The maximum power output can only be achieved when both the number of turns $N_{c}$ and the wire diameter $d_{w}$ increase while still fitting within the desired coil geometry. This ensures the optimal utilization of the available space for winding, maximizing the induced EMF and minimizing resistance losses (see Formulas (17) and (18)).

(17) $$l_{w}=2\pi a_{c}N_{c}=2\pi m\left[r_{ic}+\left(n-0.5\right)d_{w}\right]\Leftrightarrow a_{c}=k_{n}\left[r_{ic}+\left(n-0.5\right)d_{w}\right];m=k_{n}N_{c}$$

As the $k_{n}$ coefficient accounts for the distribution of turns along the coil height, which is two times the magnet length, and the following ratio defines how many horizontal turns fit relative to the vertical ones, $\left(m-n\right)/m=k_{n}$ and $n/m=c_{c}/\left(2l_{m}\right)\approx 0.1-0.15$, and the number of turns along the coil height, m was replaced in Equation (17). It is considered that n is the number of turns along the coil winding thickness $c_{c}$, and the product m·n should cover the entire winding surface $c_{c}\cdot 2l_{m}$. In conclusion, $k_{n}\in \left[0.85-0.9\right].$

(18) $$P_{cmax}=\frac{{\pi^{2}\left(\omega_{m}B_{r}k_{f}r_{ic}^{2}\right)}^{2}}{32\rho a_{c}}{\left(\sqrt{N_{c}}\cdot d_{w}\right)}^{2}$$

The coil wire diameter $d_{w}$, without insulation, can be determined with the following formula:

(19) $$d_{w}=\frac{\sqrt{8\rho a_{c}N_{c}P_{cmax}}}{U_{c}}$$

If we consider the number of turns, $N_{c}=9300\ldots 12500$, and a mean coil radius of $a_{c}=0.010\;m$, for a maximum power $P_{cmax}=2\;W$, we obtain $d_{w}=0.125\ldots 0.145\;mm$.

If the insulation is 5 to 15 µm and no geometrical constraints are considered, an enameled copper wire of 0.15 mm should be used for 9300 turns and an enameled copper wire of 0.2 mm should be used for 12,500 turns in order to obtain 2 W.

Equation (18) highlights that power increases with a larger wire diameter (reducing resistance) and a higher turn count (enhancing flux linkage), but both must be balanced within the coil’s spatial constraints. With the accurate estimations of the magnetic flux, resistance, and power, the optimal coil geometry and overall electromagnetic generator design can be effectively determined, using the magnet’s dimensions as input parameters.

### 2.3. Theoretical Modeling of the Piezoelectric–Metal Composite Structure

To simulate the voltage generated by a piezo–metal composite structure via the direct piezoelectric effect, where oscillations from the levitating magnet are transferred through magnetic repulsion to fixed end magnets (fixed to metal disks), it is necessary to evaluate both the magnetic force acting on the system and the distribution of mechanical stresses within the composite plate. The following analysis approximates the composite disk as two springs in series, representing the piezoelectric and metal layers, respectively. It assumes that a 24 mm diameter piezoelectric disk is perfectly and rigidly bonded to the top of a 41 mm diameter metal disk, with mechanical stresses uniformly distributed throughout the structure. Also, each plate contributes to the total deformation under the given axial load $F_{mag}$. From isotropic linear elasticity (no electric field yet), the radial strain component is zero (piezo clamped on the edge): $\delta_{r}=0=\left[T_{1}-\nu \left(T_{1}+T_{3}\right)\right]/Y_{p}$. This is because both plates are clamped at their edges (circumference) and the axial stress $T_{3}$ is linked to the radial stress $T_{1}$ by Poisson’s ratio ν=0.31, $T_{1}=\nu /\left(1-\nu \right)T_{3}$ (radial symmetry). The 10 mm in diameter end magnets are glued and centered on the 41 mm in diameter brass disk. The resulting hybrid piezo–metal composite forms a thin 0.42 mm disk structure. The brass disk is held at its rigid edge by a cylindrical support. A copper or brass support plate provides structural reinforcement, aiding in the fabrication of beams and other bending structures while preventing failure when plucked by magnets. The copper or brass sheet was fully bonded to the PZT beam, ensuring efficient strain transfer from the metal layer to the PZT beam. This high efficiency is attributed to the superior elasticity of the metal and the improved mechanical properties of the composite structure. Calculations show that the effective Young’s modulus of the composite structure is close to that of the metal. $Y_{p}=50\text{–}63\;GPa$ is Young’s modulus of the piezoelectric material (PZT-5H) and $Y_{m}≅120\;GPa$ is Young’s modulus of the metal, yellow brass [17,25]. The composite disk presented in this paper is a commercially available PZT−5H and yellow brass structure. The modeling of a composite piezo–metal structure and the constitutive equations for the elastic and the piezoelectric layers are presented by Xianfeng Wang et al. [26]. Other governing equations for a mechanically damped system with a central mass and segmented composite beam harvesters can be found in [27,28].

The following equations were adapted to account for tensile or compressive stress in both radial and axial directions. Displacement (i.e., strain variation) occurs primarily along the axis of the magnet’s motion. For PZT-5H, the relevant piezoelectric strain constants are $d_{33}=593\frac{pC}{N}\;{\mathrm{and}\;d}_{31}=-274\frac{pC}{N}$ [29,30].

For a moving magnet with a diameter of 10 mm and a length of 20 mm ($l_{m}$) approaching the stationary magnet at the tube end, also 10 mm in diameter but with a thickness of 2.5–3 mm ($l_{1}$), which forms part of the magnetic repulsive cushion, a correction function $f\left(x\right)=a\cdot e^{-bx}+c,$ where *x* is expressed in mm, was applied to improve the fit with data obtained from numerical simulations (error under 7%). For this configuration, specific constants were calculated, a=0.87, b=0.6, and c=0.71. The equivalent magnet length $l_{eff}=\sqrt{\left(l_{1}^{2}+l_{m}^{2}\right)/2}$ was defined as the root mean square of the two individual magnet lengths (this approach also helped in reducing the error).

(20) $$F_{mag}=f\left(x\right)\frac{B_{r}^{2}S_{m}^{2}}{{2\mu }_{0}}\cdot \frac{1}{{\left(x+l_{eff}\right)}^{2}},\;f\left(x\right)=a\cdot e^{-bx}+c$$

(21) $$D=d_{33}{T_{3}+2\left[\nu /\left(1-\nu \right)\right]\left|d_{31}\right|T_{3}+\varepsilon_{eff}E}_{eff},\;E_{eff}=U_{p}/t_{eff}\;$$

The axial magnetic force that is applied to the clamped piezoelectric composite plate is split into two components, one for the metallic disk and one for the piezoelectric disk. The axial mechanical stress $T_{3}=F_{mag}/A_{eff}$, acting on the piezoelectric disk, is concentrated on the glued magnet surface $A_{p}=S_{m}=\pi r_{m}^{2}=0.00785\;m^{2},\;r_{m}=5\;mm$. $A_{m}=0.0013\;m^{2}$ is the brass disk area. The respective stiffnesses are $K_{eff}$ (effective), $K_{p}$ (piezo), and $K_{m}$ (metal).

(22) $$\frac{F_{mag}}{K_{eff}}=F_{mag}\frac{t_{p}}{Y_{p}A_{p}}+F_{mag}\frac{t_{m}}{Y_{m}A_{m}}=\delta_{p}+\delta_{m},\;K_{p}=\frac{Y_{p}A_{p}}{t_{p}},\;K_{m}=\frac{Y_{m}A_{m}}{t_{m}}$$

**Figure 9:**
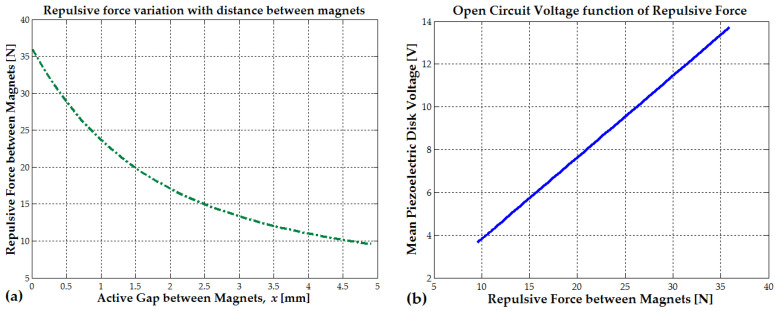
(**a**) Repulsive force variation with active gap distance between 10 mm in diameter magnets, 20 mm and 3 mm long; (**b**) Mean generated voltage of the piezoelectric disk function of magnets’ repulsive force.

The axial force acting on the composite plate will remain below the maximum repulsive magnetic force between the magnets under normal operating conditions (see Figure 9a). This axial force will exceed the magnetic repulsion force only when the magnets come into direct contact. At that point, a portion of the impact force, driven by the acceleration of the oscillating magnet and typically ranging between 700 and 2000 N, will be transmitted to the end magnets. Such high forces may cause permanent damage to the piezoelectric stack. In conclusion, the magnetic cushion also serves to protect the piezoelectric stack by mitigating excessive axial mechanical strain, displacement ($\delta_{p}\;and\;\delta_{m}$), and stress (T_3_).

The piezoceramic disk thickness was $t_{p}=0.3\;mm$ and the metallic disk thickness was $t_{m}=0.12\;mm$. Assuming two orthogonal components, axial and radial, the effective permittivity is $\varepsilon_{eff}=\varepsilon_{33}=\varepsilon_{33}\cos^{2}\varphi +\varepsilon_{31}\sin^{2}\varphi$, but here, φ=0°. The axial (z) and (x, y Cartesian coordinates) permittivity values are given by $\varepsilon_{33}=3400\cdot 8.85pF/m,\;\varepsilon_{11}=1700\cdot 8.85pF/m$.

The voltage and the effective electric field $E_{eff}$ are estimated for the open-circuit condition, where electric displacement is zero (no external charges flow) and D=0. The electric field generated internally acts to cancel out the piezoelectric contribution. Formula (22) can be combined with Formula (21), resulting in

(23) $$U_{p}=\frac{Y_{m}{\left[d_{33}+2\left(\nu /\left(1-\nu \right)\right)\left|d_{31}\right|\right]F}_{mag}}{\varepsilon_{eff}K_{eff}},\;K_{eff}=\frac{K_{p}K_{m}}{K_{p}+K_{m}}$$

To better account for the mechanical interaction between the bonded piezoelectric and metal layers, the often-used geometric ratio $t_{eff}/A_{eff}$ was replaced with a stiffness-based term $Y_{m}/K_{eff}$, where $K_{eff}$ captures the effective series stiffness of the composite structure. This leads to a more physically grounded estimation of the open-circuit voltage under combined axial and radial stress conditions.

For $F_{mag}=45\;N$, the average estimated voltage from the direct piezoelectric effect was $U_{p}=17\;V$ (hard running). For $F_{mag}=60\;N$, the mean generated voltage from the direct piezoelectric effect was $U_{p}=23\;V$. The mean piezoelectric disk voltages estimated for repulsive forces between 10 and 35 N are presented in Figure 9b.

The piezoelectric disk average voltage (peak value) from Figure 9b corresponds to the soft-jumping or soft-running case that is presented in the Experimental Section.

## 3. Results

### Hybrid Piezo–Electromagnetic Generator Measurements

By considering a design that slowly damps the central magnet’s vibration (magnetic cushion), four generators were constructed to convert low-amplitude mechanical movements, such as those generated by walking or light running, into sustained electrical energy. This approach enabled the harvesting of useful power over nearly one second, favoring continuous energy collection over intermittent bursts. Additionally, by increasing the magnet’s oscillation frequency four times beyond the typical human step and running frequencies, the system achieved more efficient energy conversion through enhanced mechanical–electromagnetic coupling. The oscillation frequency of the central magnet is influenced by the strength and length of the end magnets (which shape the magnetic restoring force), the tube length (which defines the available displacement range), and the central magnet’s mass (with heavier masses lowering and lighter ones raising the natural frequency through a mass–spring relationship). By increasing the length and strength of the top and bottom magnets, both the pushing force and the magnetic field intensity are enhanced, resulting in a larger active gap between the magnets.

Stronger repulsive forces combined with a lighter central magnet enable prolonged oscillation. Additionally, by optimizing the tube and coil dimensions, the oscillation frequency can be tuned to exceed typical human running frequencies by up to six times.

**Figure 10:**
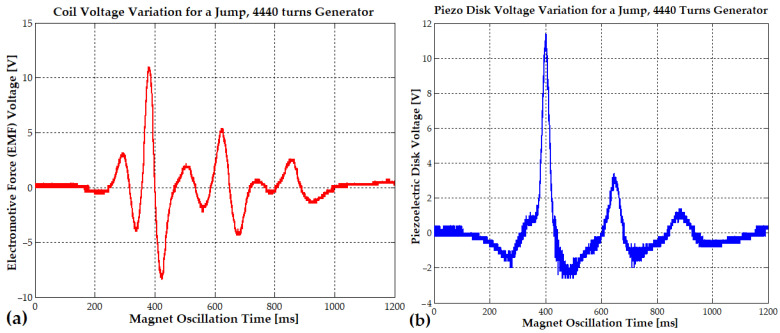
(**a**) Measured EMF of a 10 mm × 20 mm oscillating magnet inside a 4440-turns coil (PEG I generator); (**b**) measured voltage of the piezoelectric disk during magnet oscillation in the PEG I generator (when jumping or running).

The first hybrid piezo–electromagnetic generator (PEG I) was equipped with a 96 mm long tube for magnet oscillation. The oscillating magnet, measuring 10 mm × 20 mm and weighing 12 g, lacked sufficient space to sustain prolonged oscillation at this length. This first generator (PEG I) featured 4440 turns with a wire diameter of 0.2 mm. The tube and coil assembly had a total weight of 96 g. While the peak current reached 27 mA during running or jumping, the peak voltage did not exceed 12 V (see Figure 10a). The piezoelectric disk also exhibited a peak voltage of approximately 11.8 V (see Figure 10b). Due to the coil’s length of 40 mm, exactly twice the magnet’s length, an 84 mm inner pipe length, and a coil thickness of 8 mm ($c_{c}$), the magnet’s oscillation distance and frequency (23–4 Hz) were limited. Consequently, only two voltage peaks of 11.8 V and 5 V were observed within 200 ms. Very small oscillations of the magnet can significantly reduce system performance, potentially to the point where minimum useful power is generated. This can occur when the magnet is positioned outside the coil, resting on the magnetic cushion, so that only a very small part of it reaches the coil turns, and when the generated voltage is minimal, typically between 1 and 5 V.

In Figure 10a, it can be observed that from a single initial vertical jump (approximately 1 Hz) at time 0, the coil and magnet continue generating electricity autonomously (without any additional external impulse) for nearly 800 ms.

The second hybrid piezo–electromagnetic generator (PEG II) featured a 120 mm long tube for magnet oscillation. The device was 44 mm in diameter (the piezo-housing cylinders) and 140 mm in length. The total weight of the device was almost 150 g.

**Figure 11:**
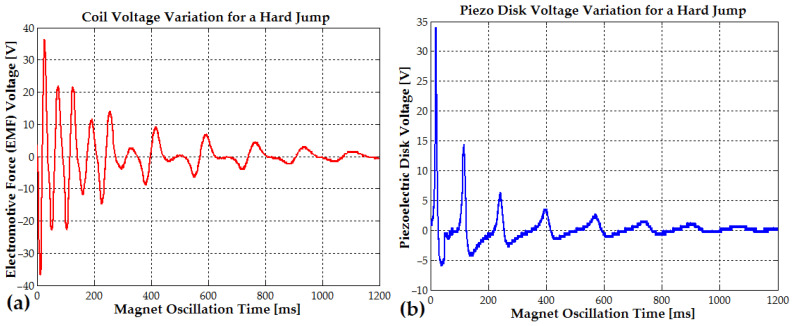
(**a**) Measured EMF of a 10 mm × 20 mm oscillating magnet inside a 9220-turns coil (PEG II generator); (**b**) measured voltage of the piezoelectric disk during magnet oscillation in the PEG II generator (when jumping or running).

During this period, the magnet exhibited a damped mechanical oscillation at an average frequency of approximately 25 Hz, corresponding to a cycle time of about 40 ms. This cycle duration was determined from Figure 11a, based on the time interval between the first and second negative and positive peaks of the induced voltage waveform.

At every 16–35 ms (32 to 15 Hz), due to slow mechanical damping, the electromotive voltage gradually decreases from a peak of 38 V to 22 V, 14 V, 9 V, 7 V, and finally 4 V. At the same time, as seen in Figure 11b, the piezoelectric disk generates a voltage that also decreases from a peak of 34 V to 14.4 V, 6.4 V, and 3.5 V with a frequency variating between 11 Hz and 6.3 Hz.

Each additional voltage pulse is stored in a ring of 5000–20,000 µF electrolytic capacitors, positioned after the parallel-connected rectifier bridges, ensuring efficient energy capture from each source and lowering the voltage to the desired level (20–24 V). The 5 V electronic regulator from the final stage charges the supercapacitors gradually over three minutes or longer (up to an hour), depending on their capacity, which ranges from 0.47 F to 11 F.

**Figure 12:**
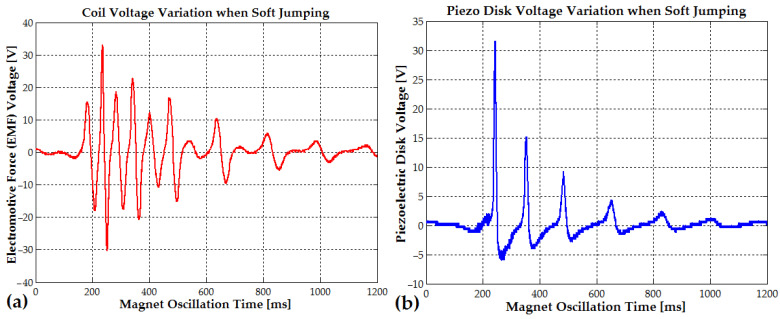
(**a**) Measured EMF (when soft jumping or hard walking) of a 10 mm × 25 mm oscillating magnet inside a 9220-turns coil (PEG II generator); (**b**) measured voltage of the piezoelectric disk in the same conditions.

**Figure 13:**
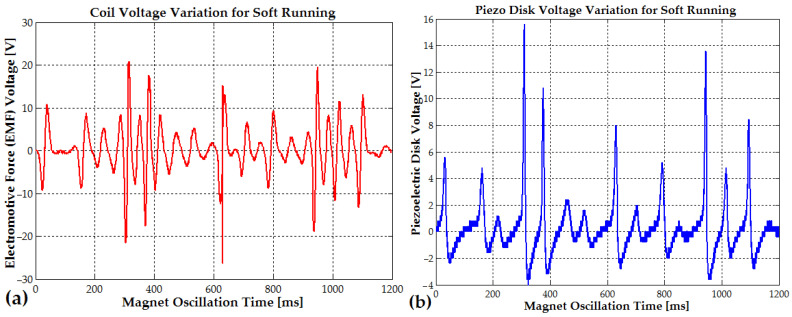
(**a**) Measured EMF (when soft running) of a 10 mm × 20 mm oscillating magnet inside a 9220-turns coil (PEG II generator); (**b**) measured voltage of the piezoelectric disk in the same conditions.

During light running or soft jumping, when the oscillation time interval between steps is reduced to approximately 300 ms (3.3 Hz), the vibration amplitude reaches 20–35 V (EMF voltage) due to the rhythmic shifts from one foot to the other (see Figure 12a and Figure 13a). In Figure 12b, the piezoelectric disk generates a voltage that also decreases from a peak of 31.6 V to 15.2 V, 9 V, and 4 V, with a frequency that slowly changes from 9 Hz to 6 Hz (the magnet slows down each time the magnet is in the upper position, outside the coil). The EMF voltage for the soft-jumping case slowly decreases from 33 V down to 10.4 V for a variation frequency between 29 Hz and 14 Hz (the magnet also slows down for each cycle).

The piezoelectric disks, activated by the direct piezoelectric effect, generated a peak voltage of approximately 32 V in the soft-jumping case (see Figure 12b). When the human subject was soft running, the voltage ranged between 14 and 16 V (see Figure 13b). The end magnets were glued at the center of the piezoceramic elements, each of which had a diameter of 24 mm. These elements were further attached to brass disks with a diameter of 40–41 mm. The resulting hybrid piezo–metal composite formed a thin disk structure with a thickness of 0.4 mm. This disk (or more disks) was glued at its circumference to a cylindrical support, allowing it to deform and oscillate along the coil and pipe axis, enabling efficient energy harvesting through bending (tensile) vibrations. A hybrid composite array consisting of two piezo–metal elements, each attached to the ends of the pipe, was also used to further enhance power generation (see Table 1). Each composite element was connected to a separate bridge rectifier composed of four Schottky diodes. The outputs of all four Schottky diode bridge rectifiers from the piezoelectric elements were connected in parallel to an electrolytic capacitor buffer for energy storage and voltage regulation.

**Figure 14:**
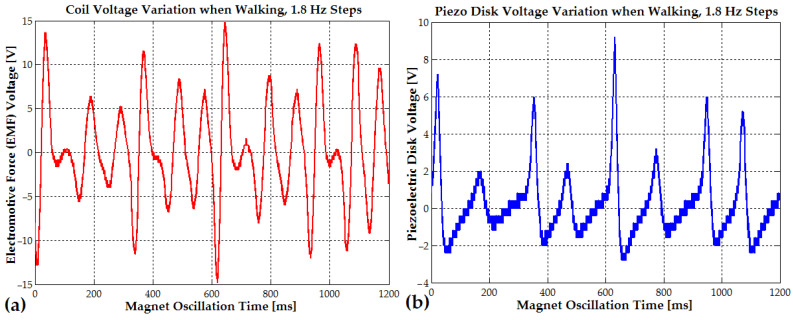
(**a**) Measured EMF (when walking) of a 10 mm × 20 mm oscillating magnet inside a 9220-turns coil (PEG II generator); (**b**) measured voltage of the piezoelectric disk when walking (PEG II).

Every 550–600 ms (the typical walking step period), the electromotive voltage gradually decreases from a peak of 14–15 V due to slow mechanical damping. It then rises again to the same value, following the rhythmic shifts from one foot to the other (see Figure 14a). The piezoelectric disks, activated by the direct piezoelectric effect, generate a peak voltage of approximately 7–9 V during walking (see Figure 14b).

The maximum power obtained from four piezoelectric disks was 23 mW when the human subject was jumping or running. In this case, the maximum voltage generated by a single piezoelectric disk (31.6 V) was comparable to the peak voltage (33 V) produced by the oscillating magnet inside the coil (see Table 1 (PEG II generator)). The piezoelectric composite structure generated only 0.63 mW in the PEG II generator during walking trials. Although this output represents just 1/21 of the power produced by the electromagnetic component, it contributes to the overall efficiency of the hybrid system. Moreover, a single element from the piezoelectric stack can function as a transducer, providing valuable data on the human subject’s movement type, gait asymmetry, and overall biomechanics. The piezoelectric power output can be further increased by incorporating more than two or four composite elements into the stack.

For the hybrid PEG I generator with 4440 turns, the coil’s internal resistance was 157 Ω, with an inductance of 145 mH. The hybrid PEG II generator, with 9220 turns, had a coil resistance of 2170 Ω and an inductance of 368 mH.

Regarding the electromagnetic generators, EG I (12,300 turns) had a coil resistance of 768 Ω and an inductance of 610 mH. Meanwhile, EG II, which had fewer windings (5070 turns), but a wire diameter of 0.3 mm, exhibited a coil resistance of 235 Ω and an inductance of 195 mH. For the EG I and EG II generators, piezoelectric stacks were not added due to large magnetic repulsion forces.

The coil is optimally designed both geometrically, having a length twice that of the magnet, and in terms of winding, with a minimum of 5000 and a maximum of 12,500 turns. The maximum generated power ranges between 0.3 and 3.0 W, while the corresponding voltage, depending on the number of turns, varies between 25 and 50 V (when running or jumping) (see Table 1).

One of the key advantages of the four generators is that the vibrations of the central magnet are damped very slowly, allowing useful electrical energy to be harvested for nearly one second. The generator is specifically designed to capture and rectify most low-amplitude vibrations, such as those produced by normal walking or light running. Rather than relying on short bursts of energy from high-amplitude vibrations, it is more effective to accumulate smaller amounts of energy over an extended period. Another advantage is the increased oscillation frequency of the magnet relative to the stepping frequency of human walking and the running frequency. This enhancement ensures a more efficient energy conversion process by optimizing the interaction between the mechanical motion and the electromagnetic induction.

**Figure 15:**
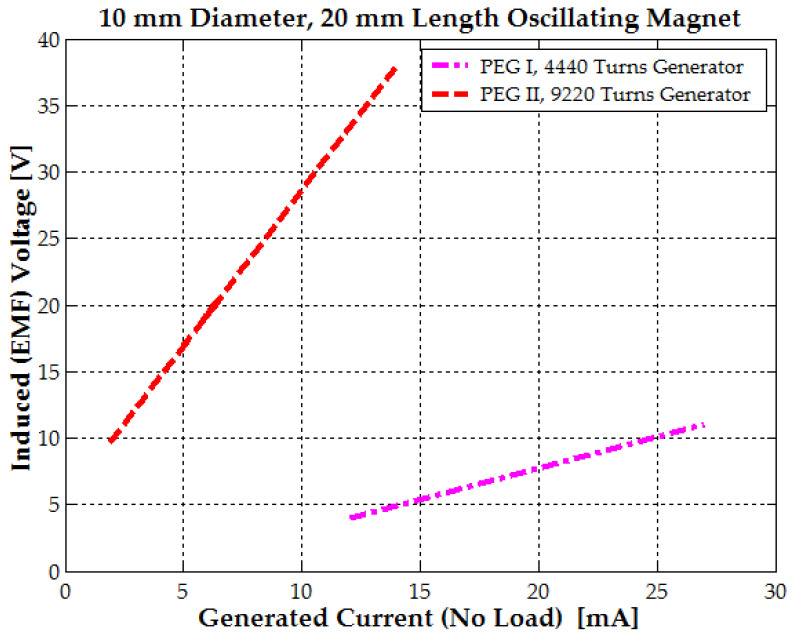
Induced voltage function of generated current for the PEG I and PEG II hybrid generators.

Because the inductance is in air and is hundreds of mH and the oscillating frequency of the magnet is low, between 8 and 25 Hz, depending on the motion type, walking, running or jumping, the inductance influence on the second Kirchhoff law can be neglected. The induced voltage drop will mainly be on the coil internal resistance and load resistance, so in consequence, the voltage variation with current for all generators will be linear (see Figure 15).

**Figure 16:**
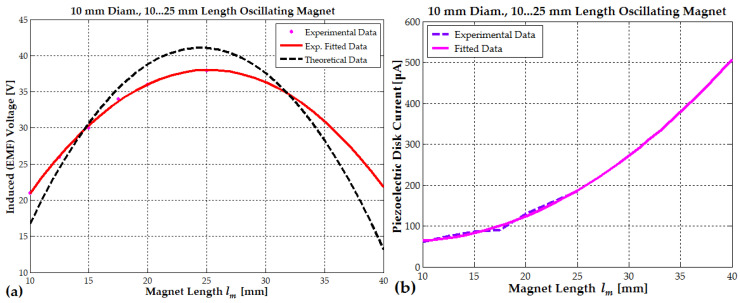
(**a**) Measured EMF of a 10 mm diameter oscillating magnet with varying magnet length during running and jumping (PEG II generator); (**b**) measured voltage of the piezoelectric disk as a function of magnet length under the same conditions.

Figure 16a shows the relationship between the induced EMF voltage and magnet length. The induced voltage increases as the magnet length increases, reaching a peak when the magnet length is approximately twice the coil length. At this point, the maximum voltage is observed, ranging between 38 V and 41 V. Beyond this optimal magnet length (20 mm for the PEG II generator), the induced voltage starts to decline. When the magnet length reaches 30 mm (5 mm beyond the optimal length), the voltage drops below 36 V. Further increasing the magnet length to 35 mm (10 mm beyond the optimal length) results in a more significant decrease, with the voltage falling below 30 V. This trend indicates that exceeding the optimal magnet length negatively impacts the induced EMF by reducing the effective interaction between the magnetic field and the coil turns. For example, the total magnet length is 30–35 mm, with 20 mm influencing the upper half of the coil and 5–10 mm affecting the lower half. As the magnetic flux increases in the upper half due to the 20 mm section, the flux in the lower half (influenced by the 5–10 mm section) decreases, partially canceling the induced EMF and leading to a net reduction in EMF.

As the magnet length increases, the magnetic force between the magnets also grows, ranging from 10 N to 35 N for a 10 mm diameter magnet. This increased force enhances the tensile effect on the piezoceramic–brass composite, resulting in greater current generation. Figure 16b shows that for a 10 mm thick magnet, the piezoelectric disk produces a current of 64 μA. When the magnet length increases to 15 mm, the current rises to 85 μA, and for a 20 mm magnet, it reaches 124 μA. At a 25 mm length, the generated current peaks at 187 μA. The PEG II hybrid generator can accommodate a central magnet of either 20 mm or 25 mm in length, with the coil length fixed at 52 mm. For a central magnet length of 25 mm, the maximum piezoelectric current generated by an array of two composites reaches 360 μA.

**Figure 17:**
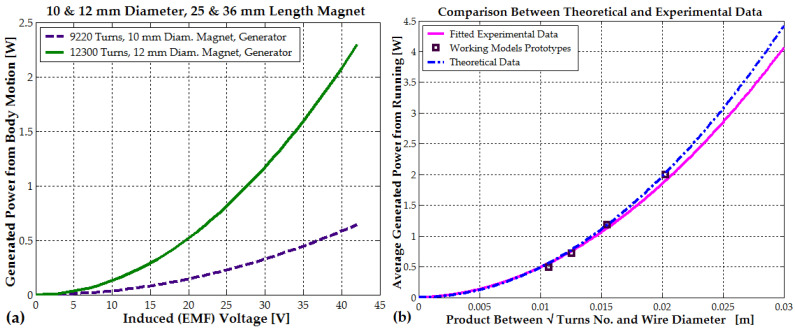
(**a**) Generated power of the PEG II and EG I electromagnetic generators as a function of induced EMF voltage (representing maximum power during running or jumping). (**b**) Average generated power of all four electromagnetic generators (and other prototypes) as a function of the product of the number of turns and wire diameter.

The PEG II and EG I electromagnetic generators represent the optimal designs for harvesting low-frequency vibrations from the human body, such as those generated by walking. The PEG II generator, with 9220 turns, achieves a maximum electromagnetic power of 0.53 W when the generated voltage reaches 38–40 V during jumping. Similarly, the EG I generator, with 12,300 turns, reaches a maximum power of 2.2–2.3 W at the same voltage range. During walking, the generated voltage is significantly lower. The PEG II generator produces 12 V, generating a power output of 36 mW. In contrast, the EG I generator achieves a voltage of 25–30 V, resulting in a power output of 1–1.17 W (see Figure 17a). When designing electromagnetic generators for maximum power output, both the number of turns and the wire diameter should be increased until the desired coil geometry is achieved and the induced EMF voltage reaches at least 10–12 V during walking (see Equations (18) and (20) and Figure 17b).

This approach ensures the optimal utilization of the available winding space, maximizes the induced EMF, and simultaneously minimizes resistance losses (see Figure 17b). A voltage between 8 and 12 V is required to power the DC/DC voltage regulator in the second stage, eliminating the need for a pre-regulator.

(24) $$P_{exp}\left(x\right)=\left(4324.44\;x^{2}+5.82\;x\right),\;where\;x=d_{w}\sqrt{N_{c}}$$

For small wires up to 0.2 mm in diameter, the bare copper diameter can be approximated as the total insulated diameter minus 12 to 20 µm. A simple formula to estimate the insulated wire diameter up to 0.25 mm is $d_{iw}=1.1\cdot d_{w}^{0.99}\;[mm]$. For the PEG II model with 9220 turns, this gives x≈0.0107 m; for the EG I generator with 12,300 turns x≈0.0155 m and for the EG II generator with 5070 turns, x≈0.02. However, the first realized generator, with 4440 turns and x≈0.011 m, did not conform to the performance trend. It only produced 0.2–0.3 W, indicating that a significant portion of the winding was ineffective. This suggests that nearly half of the turns contributed negligible power due to low flux coupling or wrong placement within the magnetic field profile.

For all other generators, the wire diameter was optimally selected based on the number of turns, ranging between 0.12 and 0.3 mm, ensuring compliance with the required geometry and size constraints. According to the previous statement, a generalized power chart (that could be used for future coil designs) from Figure 17b is presented.

The coil was optimally designed both geometrically, with a length twice that of the magnet, and in terms of winding space, with turns ranging from a minimum of 5000 to a maximum of 12,500. It was observed that the coil winding should not extend more than 6 mm in the radial direction.

For all prototypes, the generated power varied between 0.3 and 3.0 W, while the corresponding voltage, depending on the number of turns, ranged from 25 to 50 V (for intensive running).

**Figure 18:**
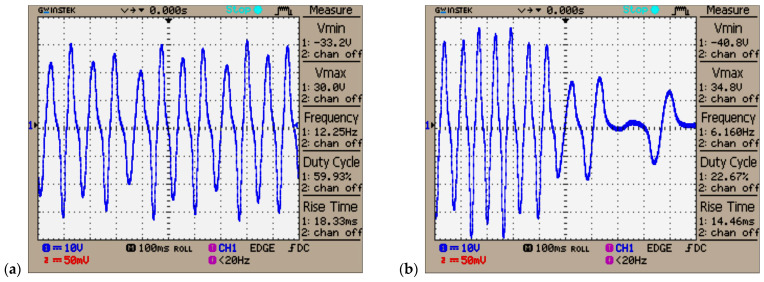
(**a**) Measured EMF (when walking or slow running) of a 12 mm × 36 mm oscillating magnet inside a 12,300-turns coil (EG I generator); (**b**) measured EMF (when jumping) of the EG I generator in the same conditions.

As seen in Figure 18a, a peak EMF voltage of 30 V is generated during walking or slow running. Similarly, Figure 18b shows a peak EMF voltage of 40.8 V when the human subject is jumping or running at a higher intensity. The hybrid piezo-inductive generators were built using the largest available piezoceramic disks: a 24 mm piezoelectric disk glued on a 41 mm brass disk, with a total thickness of up to 0.42 mm (0.1–0.2 mm for the brass and 0.3 mm for the piezo). The central magnet with N48 remanence (20 or 25 mm in length, with a repulsive force of $F_{1}=43-45\;N$) and magnets with N42 remanence (positioned at the ends, 2.5 mm in length, with a repulsive force of $F_{2}=16\;N$), each with a 10 mm diameter, generates a combined repulsive force of approximately 25–35 N between a 0.1 and 1 mm distance, as seen in Figure 9a and by using Formula (20).

**Figure 19:**
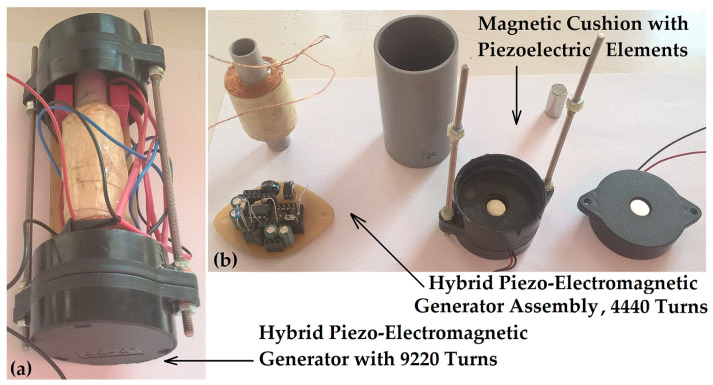
(**a**) Hybrid piezo–electromagnetic generator PEG II (9220 turns)—practical realization for human motion energy harvesting; (**b**) PEG I generator (4440 turns)—first practical model.

Experimentally measured voltages of 25 V (with a 20 mm central magnet, under soft-jumping conditions) and 31 V (with a 25 mm central magnet, also under soft-jumping conditions; see Figure 12a) support the hypothesis that a longer magnet and stronger repulsive force enhance the overall efficiency of the device. The experimental setup used to obtain these results is shown in Figure 19a and detailed in Table 1. The first prototype of the electromagnetic generator had a radial build-up thickness of approximately 8 mm; however, nearly half of this thickness contributed minimally to EMF generation, as illustrated in Figure 19b.

**Figure 20:**
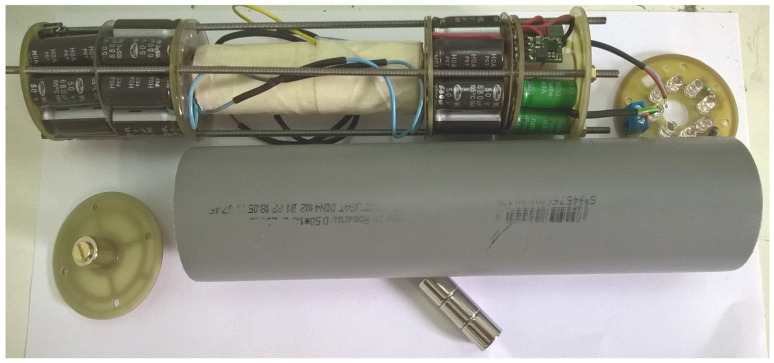
Realization of the electromagnetic generator EG I with 12,300 turns and a 12 mm × 36 mm oscillating magnet.

The ring magnet, with an outer diameter of 12 mm, an inner diameter of 5 mm, and a height of 12 mm, is a neodymium magnet with N42 magnetization and an adhesive force of approximately 4.4 kg. The 5 mm central hole serves to reduce air damping and decrease the overall weight of the magnet, allowing for an extended magnet length of 36 mm (see Figure 20).

The central oscillating magnet was created by stacking three identical 12 × 12 mm magnets together. Additionally, the 5 mm hole can be used to secure the repelling magnets to the corresponding end caps. The central magnet, with a diameter of 12 mm, exerts a repulsive force of approximately 120 N. The opposing magnet in the magnetic cushion section, also sized 12 × 12 mm, exerts a counteracting force of about 42 N. The resulting net repulsive force between the two magnets is therefore approximately 63 N, calculated using the correction function $f\left(x\right)=6.88\cdot e^{-0.475x}+2.84$ and the magnetic force expression from Equation (20). To enhance the oscillation frequency, particularly in response to the increased length of the inner pipe, a larger repelling magnet was selected for the end caps. This design choice helps to sustain the amplified oscillatory motion within the extended structure. Given that the coil length is 82 mm, an additional 40 mm of space is required on both sides, along with the length of the end magnets. Therefore, the total inner pipe length should range between 185 mm and 200 mm. A length of 196 mm was used for the construction of the EG I generator. The cylindrical shell has an outer diameter of 51 mm, and this third device has a total weight of 460 g.

**Figure 21:**
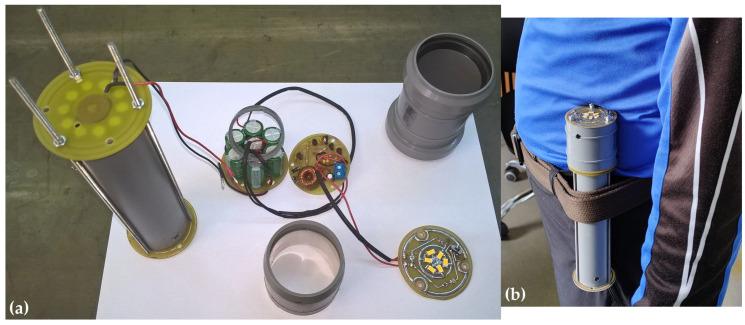
(**a**) Realization of the electromagnetic generator EG II with 5070 turns and a 15 mm × 25 mm oscillating magnet, the pre-regulator (trough hole mounting version), and a part of the electronic management system is presented; (**b**) the same device is attached to the waist (torso) using a belt.

Figure 21a,b presents an electromagnetic generator (EG II) capable of instantly producing at least 2 W during movement, either from running or the pressure exerted by exhalation. The model was tested using a magnet with a 15 mm diameter and 25 mm length, sliding inside a PVC tube with an 18 mm outer diameter. The inner tube has a length of 154 mm, and the smaller shell diameter is 43 mm. An additional housing tube, used for electronics, measures 54 mm in diameter and 58 mm in length. The total weight of the device is 542 g. The winding wire used has a diameter of 0.3 mm. It is recommended to increase the wire diameter as the magnet’s radius increases. The latest prototype was relatively bulky, which reduced the magnet’s oscillation when walking. This lower oscillation amplitude could also be attributed to air damping, combined with the opposing force generated by the induced current’s magnetic field. This opposing field either repels the magnet from the coil or slows its descent. Since the coil current reaches 100 mA, this deceleration effect is significant, making it difficult for the user (at some point) to further increase the vibration amplitude and frequency.

As a result, the EG II generator is most effective for energy harvesting only during intense running or jumping, when the oscillating force is much greater than the opposing force.

Hard walking and gait asymmetry, where the length or force of each step differs slightly between legs, can be detected using piezo–metal composite disks. For example, in Figure 14b, the voltage amplitude differs between steps, measuring 7.2 V for one step and 9.2 V for the other. Similarly, during soft running (Figure 13b), the piezoelectric disk recorded voltage amplitudes of 15.8 V and 13.8 V for alternating steps.

A significant voltage difference during walking or running may indicate improper posture, which could eventually lead to chronic pain or arthritis. Studies suggest that the average walking asymmetry in healthy individuals ranges from approximately 5% to a maximum of 15%. Any asymmetry exceeding 20% may indicate underlying health issues [31,32,33,34]. This device could be directly attached to an athlete’s waist for monitoring step frequency and strength variations while running, without the need for specialized shoes or other wireless equipment.

The EG I and EG II electromagnetic generators were unable to effectively utilize the power generated by the piezoelectric disks due to the significant forces (that can damage a single or two composite elements), ranging from 72 to 100 N, between the magnets. A potential solution is to fabricate a longer piezo–metal array composed of 8 to 16 composite disks, capable of withstanding these forces and generating usable power. Alternatively, using thicker composite plates could also address the issue.

The hybrid generator can be equipped with piezotronic devices (i.e., piezotronics) that utilize the piezoelectric potential to specifically adjust the carrier transport properties at the metal−semiconductor contact interface and can be attached to the piezo–metal disks or flexible ones directly to the body to sense all body movements [35].

Piezopotential is an electric field generated in noncentrosymmetric crystals under strain, caused by immobile ionic charges. In asymmetric semiconductor devices, tensile strain creates a negative piezopotential that repels electrons from the p−n junction and increases the local Schottky barrier height. Compressive strain, in contrast, generates a positive piezopotential that attracts electrons toward the metal−semiconductor interface or p−n junction, reducing the Schottky barrier height. This principle, central to piezotronics, extends beyond simple strain-gated devices to include strain-controlled transistors functionalized by piezopotential [36].

For electronic modules without SMD components, the cylindrical or ring-type converter that attaches to the generator consists of four main modules. The first ring-type rectifier module with Schottky diodes (SB140 or SB160) uses SB140 for voltage intervals of 25–35 V and SB160 for voltages up to 50 V. The electrolytic capacitors are arranged in a circular layout across multiple levels, depending on the available volume. An example of a ring-type rectifier PCB is shown in Figure 20. The large capacitive buffer (5000 to 20,000 µF) has the role to limit the voltage to the desired input value (from 40–50 V to 20–30 V) and to be the first charging stage. The second switching pre-regulator module is used to further step down the rectified voltage from 20 to 30 V, given by the electrolytic capacitor buffer, to a range of 7–12 V. The third switching voltage regulator module used for a 5 V output (e.g., Pololu Step-Down Voltage Regulator, D24V5F5) provides a stable 5 V output, suitable for safely charging the supercapacitors and for connecting to standard USB-powered devices or other connectors. The fourth disk-shaped (ring) energy storage module with supercapacitors can be combined with the rectifier module using electrolytic capacitors to maximize the use of the cylindrical space. See Figure 21a for the 33 F, 5 V supercapacitor example. The fifth module, the LED lighting unit, is equipped with a three-way mini switch that allows it to either disconnect from the supercapacitors, connect to them, or connect to a first-stage rapid charging circuit made of electrolytic capacitors (used for emergency lighting, with separate LEDs). The module also includes a “dual LED flasher” or “firefly”-type oscillating circuit operating at a frequency between 60 and 100 Hz. See Figure 21a,b for an example of the SMD LED assembly.

The separate electronic circuits and supercapacitors form a complete backup power source. This source can provide light at the end of the day for about 3 min (with a 0.2–0.47 F supercapacitor) or up to 1 h (depending on the brightness level and by using a bigger 11 F supercapacitor). It can also power devices such as a radio, a GPS module, or even a mobile phone via the USB port and a dedicated 5 V output connector. Using a supercapacitor larger than 11 F is not recommended if the power source is intended for daytime use, as the charging time at 5 V can become quite long, 30 min and up to 1 h, depending on the vibration amplitude. The charging time was determined on a maximum power output of 2–3 W. For the typical human activity (such as walking or hiking), the power rarely exceeds 1.5 W peaks, and the full charging will take more than one hour.

For example, a 33 F supercapacitor built from six units connected in pairs (to allow for 5 V operation) required over 3 h to fully charge. To achieve faster charging, a different strategy should be implemented. This involves using a three-stage charging system combined with an energy management circuit. The first stage uses electrolytic capacitors for rapid initial energy storage. The second stage operates at 5 V with smaller supercapacitors (1 to 5 F) to handle intermediate buffering. Finally, the third stage consists of large 33 F supercapacitors for long-term energy storage.

Assuming the generator is used during a mountain hike, with an average vibration frequency of 1.5–1.8 Hz over a period of 4–5 h, the average power generated would be around 1 to 1.5 W. This results in approximately 20,000 J of energy (1.4 W × 14,400 s ≈ 20,000 J). To store this energy at 5 V, a supercapacitor with a capacity of about 1600 F would be required. However, in practice, charging such a large supercapacitor would be extremely slow, making 33 F supercapacitors a much more practical and suitable choice in this scenario.

**Figure A3:**
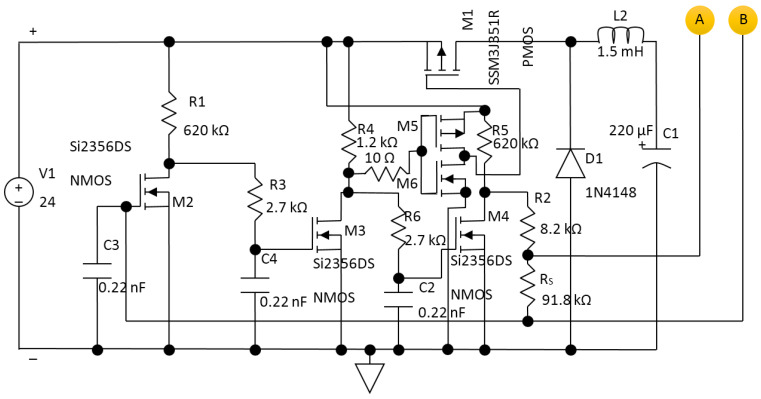
The electronic diagram of the voltage pre-regulator, with a variable 20–70% duty cycle.

**Figure A6:**
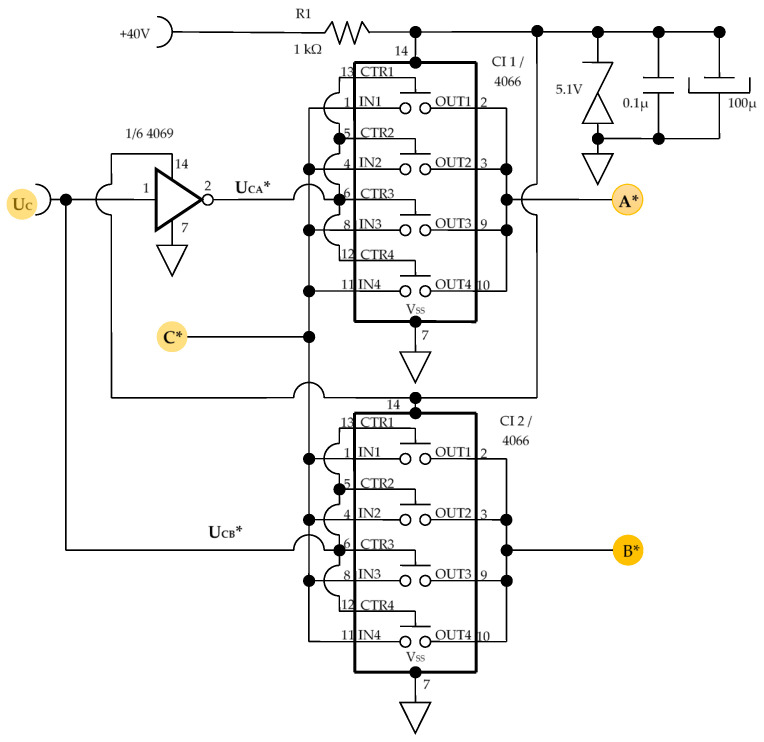
Diagram of automatic electronic switching across different operational modes (with or without pre-regulator).

**Figure A7:**
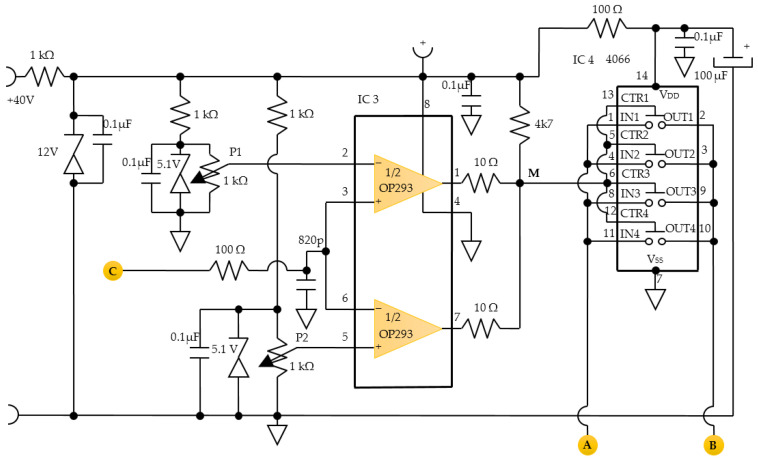
The electronic block for switching the duty cycle function of input voltage.

The pre-regulator, through-hole mounting version, is shown in Figure 21a. This version has a fixed duty cycle of 0.5. Its purpose is to further reduce the voltage output of the electrolytic capacitor buffer, which ranges from 15 V to 24 V, depending on the type of movement, to between 7.5 V and 12 V. To implement the energy management system, including the SMD version of the pre-regulator (with a two-step variable duty cycle), the circuit shown in Appendix A (Figure A3) was simulated in LTSpice and used to design a printed circuit board (PCB) populated with surface-mount (SMD) components. The entire circuit was finalized with the electronic switching components presented in Figure A6 and Figure A7 to complete the energy management system.

## 4. Discussion

The hybrid piezo–electromagnetic generator presented in this study demonstrates a highly optimized approach to harvesting energy from low-frequency human body movements, such as walking, running, or jumping. Among the various configurations tested, the best coil geometry was found to involve a coil length slightly greater than twice the length of the oscillating magnet. This configuration maximized magnetic flux linkage, ensuring that the magnet’s movement across the coil induced a significant and efficient electromotive force (EMF). Furthermore, it was observed that the coil’s radial thickness should not exceed the magnet’s radius to ensure that all turns are effectively exposed to the changing magnetic flux. To maintain compactness and efficiency, the coil’s radial build-up should be limited to 6 mm or less because the excess thickness leads to diminished induced voltage, as outer layers experience weaker magnetic fields, under 0.2 T.

In terms of induced voltage, the EMF generated by the electromagnetic coil was directly influenced by the relative position and movement of the magnet within the coil. The EMF reached its peak when the magnet was halfway inserted into the coil, maximizing the rate of flux change. For walking, peak voltages ranged from 12 to 30 V, while running or jumping generated voltages as high as 42 V (for a 12 mm in diameter magnet). The precise voltage output depended on the number of coil turns, the magnet size, and the oscillation frequency, which itself was amplified by using a magnetic repulsion cushion. This cushion effectively multiplied the body’s vibration frequency, enhancing the rate of magnetic flux change and improving energy conversion.

Magnetic flux through the coil was most efficiently harnessed when the magnet’s leading end approached the coil center with a minimal air gap. Simulations showed an average magnetic field of approximately 0.71 Tesla at a 0.2 mm gap for a 10 mm × 20 mm cylindrical magnet. Importantly, this study emphasizes that the magnet’s length has a greater impact on the generated power than its diameter. An optimal balance was achieved when the magnet’s length was nearly half the coil’s length; longer magnets showed diminishing returns due to flux cancellation across the coil.

A particularly useful metric highlighted in the research was the product of the square root of the number of turns and wire diameter, which served as a reliable indicator of coil performance. Higher values of this product correlated strongly with a higher output power, following a second-order (quadratic) relationship. This research also showed that the internal resistance remained manageable and coil geometry constraints were met. For example, the EG I generator, using 12,300 turns of a 0.15 mm wire, achieved power outputs above 2 W. Conversely, coils with low turn counts or excessive radial thickness performed poorly due to inefficient magnetic coupling and increased resistance.

One of the key advantages of the four generators is that the vibrations of the central magnet are damped very slowly, allowing useful electrical energy to be harvested for nearly one second. The generators are specifically designed to capture and rectify most low-amplitude vibrations, such as those produced by normal walking or light running. Rather than relying on short bursts of energy from high-amplitude vibrations, it is more effective to accumulate smaller amounts of energy over an extended period. Another advantage is the increased oscillation frequency of the magnet relative to the stepping frequency of human walking and the running frequency. This enhancement ensures a more efficient energy conversion process by optimizing the interaction between the mechanical motion and the electromagnetic induction.

The PEG II and EG I generators emerged as the most effective designs. PEG II, with 9220 turns of a 0.12 mm wire, offered a balanced trade-off between size, voltage output, and power efficiency, achieving up to 0.53 W. The EG I, with 12,300 turns and a 0.15 mm wire, reached up to 2.2 W during vigorous motion. Both models demonstrated that effective energy harvesting from human motion is possible with the careful optimization of the coil geometry, magnetic configuration, and electronic regulation. The modular design and efficient voltage regulation architecture further enhance the system’s applicability for wearable electronics, remote sensors, or emergency lighting.

Because the magnetic field generated by the induced current creates an opposing force that slows the magnet down, increasing the voltage up to the 40 V limit would improve efficiency. This voltage limit is determined by the use of discrete transistors rated for 40 V or 60 V, as well as compact electrolytic capacitors rated at 50 V.

The energy harvested from the hybrid system was managed through a multi-stage electronic circuit design, ensuring both efficiency and practical usability. The first stage involved Schottky diode bridges and large electrolytic capacitors (5000–20,000 µF), which acted as a buffer to capture high-voltage pulses and smooth the input. A second-stage switching pre-regulator stepped the voltage down to 6–12 V, followed by a highly efficient 5 V switching buck converter (such as the Pololu D24V5F5), which provided power for USB devices, lighting modules, or sensors. Storage was handled by supercapacitors, with optimal configurations using 1–5 F for intermediate buffering and between 11 F and 33 F for extended operation. The Pololu D24V5F5 converter maintains an efficiency of up to 92% when the input voltage is kept below 12 V, contributing to overall energy optimization.

The large electrolytic capacitor buffer inherently limited the voltage without requiring a pre-regulator. Since the magnet’s oscillation frequency was much higher than the capacitor’s charging time, the capacitor did not have enough time to fully charge. As a result, the voltage typically peaked at only 20–30 V, rather than reaching the 40 V maximum. Nevertheless, the generated energy was still effectively stored through the accumulation of charge within the buffer. This method of passive voltage limitation avoided the need for additional circuitry but involved a trade-off between the overall device weight, available space, and achievable voltage level. The switching pre-regulator was still required to further reduce the voltage, initially by half, by setting the duty cycle to 0.5 when the input voltage was within the 12 V to 20 V range. For pre-regulator input voltages exceeding 21 V, the voltage was reduced even further, by approximately one-third, using a duty cycle of 0.33 or 0.38.

Due to unpredictable body movements and to ensure efficient energy flow, the system automatically disconnected the pre-regulator from the hybrid generator whenever the voltage dropped below 12 V, using the MC14066B SOIC variant for switching control. This energy management strategy was implemented through the circuit design shown in Appendix A.

## 5. Conclusions

Design optimization revealed that the most efficient coil geometry uses a length slightly more than twice the magnet’s length and a radial build of under 6 mm, avoiding flux inefficiencies. Longer magnets showed diminishing returns due to flux cancellation across the coil. One of the key advantages of the four generators is that the vibrations of the central magnet are damped very slowly, starting from a peak frequency of 8 to 25 Hz (depending on the body movement type), due to the magnetic cushion, and allowing useful electrical energy to be harvested for nearly one second.

Another key finding is that the square root of the coil turns times the wire diameter predicts output power with a quadratic relationship. For instance, the EG I model, with 12,300 turns of a 0.15 mm wire, reached 2.2 W, while PEG II balanced compactness and performance at 0.53 W. To accommodate unpredictable body movements (from walking to running) and maintain efficient energy flow, this paper proposes an automated voltage regulation mechanism. Using the MC14066B SOIC switch, the pre-regulator is automatically disconnected whenever the input voltage drops below 12 V, preserving energy and maintaining efficiency. The voltage is reduced through a three-stage regulation system, an electrolytic capacitor buffer limits voltage peaks to 15–30 V, and then a switching pre-regulator steps the voltage down to 6–12 V while preserving ~92% efficiency. The buck converter, from the final stage, delivers a stable 5 V for charging supercapacitors. This staged energy management approach ensures reliable power delivery and system stability, as detailed in Appendix A. The device combines mechanical and electrical design optimization with modular scalability. The electromagnetic output can be scaled to 5 W by adjusting coil turns and geometry, while the piezoelectric module can reach 0.3 W by stacking 16 composite elements. Modular configurations allow multiple units to operate in tandem, supporting total outputs of up to 10 W.

The generator can also function as a biomechanical transducer. Piezoelectric elements can detect gait asymmetry, movement patterns, and posture deviations. This dual-use capability enhances its applicability in wearable tech, medical diagnostics, and motion tracking systems. By enabling long-duration, low-amplitude energy capture, the proposed hybrid generator offers a scalable and practical solution for powering wearable electronics, remote sensors, and energy-autonomous systems, eliminating reliance on batteries and extending device functionality through integrated sensing. Hybrid generators can be adapted to harvest energy from human breathing using a two-way valve system. One valve opens during exhalation to direct airflow through the generator, while the other remains closed, reversing roles during inhalation to allow continuous energy capture. An alternative design uses reed valves, which respond only to pressure gradients, or reed switches paired with a center magnet end position for precise, magnetically controlled valve actuation.

## Figures and Tables

**Table 1:** Technical specifications of the four hybrids piezo–electromagnetic generators, and the functions of the body type movement.

Body Motion Type	Technical Specifications	PEG I, 4440 Turns 0.2 mm Wire	PEG II, 9220 Turns 0.12 mm Wire	EG I, 12,300 Turns 0.15 mm Wire	EG II, 5070 Turns 0.3 mm Wire
Walking	Coil Voltage [V]	4	12	30	16
	Coil Current [mA]	12	3	39	68
	Piezo Voltage [V]	3	5	-	-
	Piezo Current [mA]	0.040	0.125	-	-
	Total Power [W]	0.049	0.037	1.17	1.09
Running or Jumping	Coil Voltage [V]	11	33	42	25
	Coil Current [mA]	27	15	53	100
	Piezo Voltage [V]	13	31.6	-	-
	Piezo Current [mA]	0.2	0.72	-	-
	Total Power [W]	0.295	0.533	2.22	2.5

**Figure A1:**
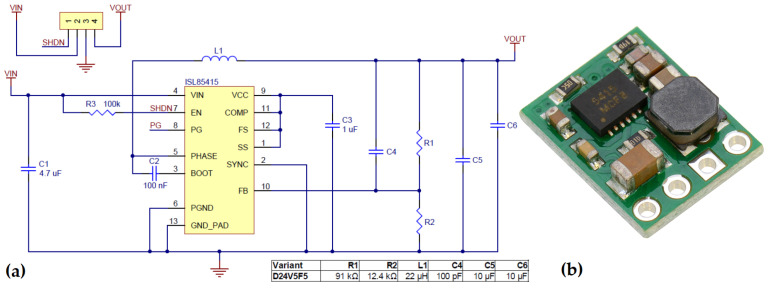
(**a**) Pololu D24V5F5 model, 5 V Voltage Regulator Electrical Scheme; (**b**) Pololu D24V5F5 model, 5 V Voltage Regulator PCB 13 × 10 mm board [37].

**Figure A2:**
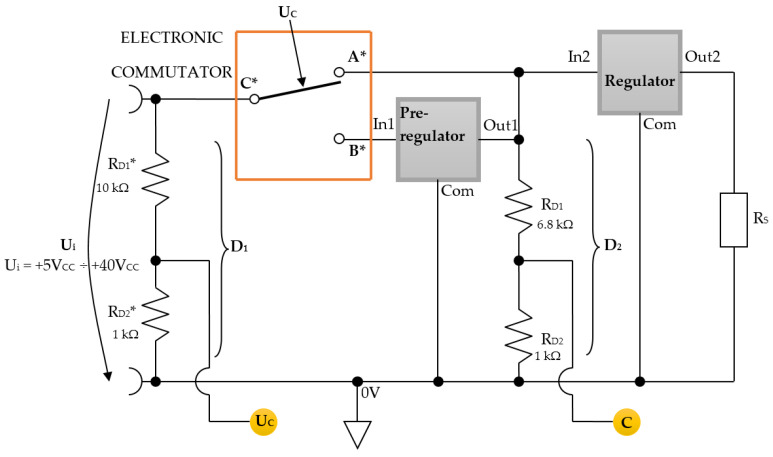
Block diagram of the energy management system implemented with or without a pre-regulator.

**Figure A4:**
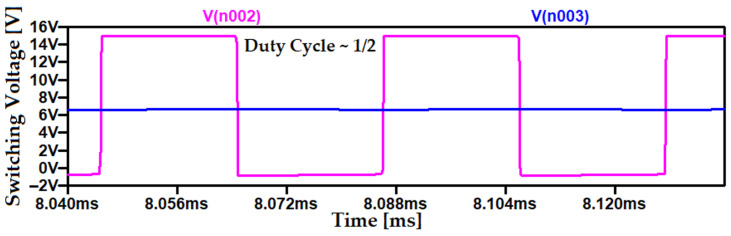
The electronic diagram of the voltage pre-regulator, with a 50% duty cycle.

**Figure A5:**
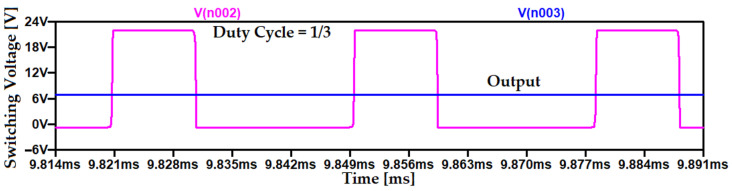
The electronic diagram of the voltage pre-regulator, with a 34% duty cycle.

## Data Availability

The original contributions presented in the study are included in the article, further inquiries can be directed to the corresponding author.

## Appendix A

Many voltage regulators were tested, like linear ones with MAX666, but the most efficient option for a voltage regulator is a switching regulator operating at a frequency of 500 kHz. This high switching frequency allows for the miniaturization of passive components, enabling the use of a smaller inductor and capacitor.

The Pololu D24V5F5 step-down (buck) regulator is a suitable choice, offering an efficiency of 90–92% when the input voltage is kept below 12 V (see Figure A1 [37]).

The pre-regulator was placed before the 5 V voltage regulator to step down the input voltage, either by a fixed ratio of 2:1 (using a fixed duty cycle of 0.5) or by employing a variable duty cycle between 0.3 and 0.5. The fixed duty cycle approach was effective only during high-amplitude vibrations, such as jumping or running. However, under low-vibration conditions like walking, the generated voltage often dropped below 12 V, causing the final regulation stage to stop functioning due to insufficient input voltage (below 5.5 V). To ensure reliable operation under all conditions, whether low or high vibrations, an adaptive energy management circuit was introduced. This circuit automatically adjusted the duty cycle as needed and disconnected the pre-regulator entirely when the input voltage fell below 12 V, allowing the system to function effectively across a wider range of input conditions (see Figure A2).

Automatic disconnection of the pre-regulator was achieved using two MC14066B SOIC SMD integrated circuits, each incorporating four switches.

The pre-regulator is composed of three main blocks: a buck converter, a ring oscillator, and an energy flow management unit (see Figure A2 and Figure A3). The buck converter is a classical step-down voltage regulator built with a Toshiba SSM3J351R PMOS transistor (SOIC SMD), a 1.5 mH SMD inductor, a Vishay 1N4148W fast-switching diode (rated 300–500 mA, SOD-123 package), and a 220 µF/50 V polymer capacitor from KYOCERA AVX. The PMOS gate is driven by a simple three-stage ring oscillator constructed using Vishay Si2356DS NMOS transistors. The energy flow management block consists of an electronic commutator made from two OnSemi MC14066B SOIC SMD ICs (Figure A6) and a voltage duty cycle control circuit implemented with an Analog Devices OP293 precision op-amp configured as a window comparator (Figure A7). A detailed analysis of the energy flow management block, which regulates operation across three distinct voltage conditions, is provided in Appendix A. The fast-switching diode used in the buck converter can be upgraded to a more efficient alternative, the Nexperia PMEG6010CEH Schottky diode, which offers significantly lower forward voltage (0.27–0.35 V) in the 10–50 mA current range, resulting in reduced conduction losses. Additionally, the full-bridge rectifiers can be replaced with the same compact, flat-lead PMEG6010CEH device to further minimize power losses and improve overall efficiency.

The power circuit includes two PMOS transistors, M1 and M5. M1 acts as the main switching transistor, while M5, along with the complementary Si2356DS NMOS transistor, functions as an inverter gate. The other transistors, M2, M3, and M4, operate as ring oscillators, in conjunction with passive components, including fixed capacitors C2, C3, and C4, and resistors R2, Rs, R3, and R6, which determine the oscillation frequency.

The minimum oscillation period of the three-stage ring oscillator can be theoretically estimated using the formula $T=6\;R_{3}C_{3}$. For $C_{3}=0.22\;nF,\;R_{3}=2.7\;k\Omega ,$ the theoretical period is T=3.5 μs. However, in practical implementations and LTspice simulations, additional switching delays, primarily due to gate propagation and parasitic capacitances, introduce timing jitter, resulting in observed oscillation periods of up to 20–30 μs. This added delay can also be influenced by NMOS threshold voltage variations and their associated parasitic effects. The voltage pre-regulator circuit was optimized for low-frequency body motion energy harvesting. As the voltage amplitude decreases, the current draw is expected to fall below 50 mA. A 1.5 mH inductor was used to reduce the current ripple across a wide current range (10–100 mA), achieving ripple as low as 0.1 mA.

According to the buck converter theory, lower load currents require larger inductance values to maintain low ripple, especially when operating at lower switching frequencies. A regulation frequency of 30–50 kHz was found suitable for this application. To accommodate even lower current levels and to provide the possibility to lower the inductance under 1 mH, the oscillation frequency can be increased to ~100 kHz by selecting $C_{3}=0.1\;nF,\;R_{3}=1\;k\Omega$.

In Figure A3, timing jitter and power losses can be further minimized by eliminating resistive elements from the diagram and instead adding complementary PMOS transistors to construct inverter gates. This approach enables reliable operation at elevated supply voltages exceeding 20 V. The current CMOS inverter gate configuration is not well suited for elevated supply voltages exceeding 20 V. Standard CMOS technology is typically optimized for lower voltage operation, usually in the range of 1.8 V to 15 V, due to the breakdown voltage limitations of the gate oxide and junctions. To enable operation at voltages above 20 V, specialized high-voltage CMOS (HV-CMOS) or LDMOS (Laterally Diffused MOS) technologies are typically required. These use thicker gate oxides and optimized doping profiles to withstand higher electric fields.

Figure A2 shows the block diagram of the electronic device responsible for managing the voltage sources. The following scenarios can describe the operation of the device:

Case 1: The input voltage is below 12 V.

If the input voltage U_i_, as shown in Figure A2, drops below the 12 V threshold, typically occurring when the human body is in motion, walking, then the voltage U_c_, derived via voltage divider D1, also drops below 1.2 V. Under these conditions, the position of the electronic switch remains as illustrated in Figure A2.

This happens because U_c_ is below the minimum input voltage required to control the 4066 integrated circuit. As a result, CI 1 is activated while CI 2 remains inactive (Figure A6). In this case, the pre-regulator is bypassed, and the electric potential at point C, determined via voltage divider D2, stays below the 1.7 V threshold (Figure A2 and Figure A7). The supply voltage from the hybrid generator is routed directly to the input of the regulator that charges the supercapacitors.

Case 2: The input voltage is between 12 V and 20 V.

If the input voltage U_i_ lies between 12 V and 20 V (Figure A2), then the voltage U_c_, derived from divider D1, falls within the 1.2 V–2 V range. As a result, the position of the electronic switch changes to include the pre-regulator before the main regulator (Figure A2). At the same time, the lower threshold of the window comparator input, set using potentiometer P1 (Figure A6), is exceeded, preventing a galvanic connection between points A and B (Figure A6). Consequently, resistor R_S_ is not short-circuited (Figure A3), and the resulting duty cycle for the combined resistance of R_2_ + R_S_ = 100 kΩ is k = 50%, (Figure A4). The output voltage from the pre-regulator remains within the 6.5 V–7.5 V range, when the input voltage is 15 V.

Case 3: The input voltage is above 20 V.

If the input voltage U_i_ exceeds 20 V (Figure A2), then the resulting U_c_ from divider D1 also rises above 2 V. Again, the switch position ensures that the pre-regulator is inserted before the main regulator (Figure A2).

Additionally, the upper threshold of the window comparator, set via potentiometer P2 (Figure A6), is crossed, which causes a galvanic connection between points A and B to form (Figure A6).

As a result, resistor R_S_ is short-circuited (Figure A3), and the corresponding duty cycle, for R_2_ = 8.2 kΩ, is k = 34% (Figure A5). The pre-regulator output voltage continues to stay within the 6.5 V–7.5 V range when the input voltage is 22 V.
